# Human *In Silico* Drug Trials Demonstrate Higher Accuracy than Animal Models in Predicting Clinical Pro-Arrhythmic Cardiotoxicity

**DOI:** 10.3389/fphys.2017.00668

**Published:** 2017-09-12

**Authors:** Elisa Passini, Oliver J. Britton, Hua Rong Lu, Jutta Rohrbacher, An N. Hermans, David J. Gallacher, Robert J. H. Greig, Alfonso Bueno-Orovio, Blanca Rodriguez

**Affiliations:** ^1^Computational Cardiovascular Science Group, Department of Computer Science, University of Oxford Oxford, United Kingdom; ^2^Global Safety, Pharmacology, Discovery Sciences, Janssen Research and Development, Janssen Pharmaceutica NV Beerse, Belgium; ^3^Oxford Computer Consultants Ltd. Oxford, United Kingdom

**Keywords:** *in silico* drug trials, drug safety, drug cardiotoxicity, Torsade de Pointes, computer models, human ventricular action potential

## Abstract

Early prediction of cardiotoxicity is critical for drug development. Current animal models raise ethical and translational questions, and have limited accuracy in clinical risk prediction. Human-based computer models constitute a fast, cheap and potentially effective alternative to experimental assays, also facilitating translation to human. Key challenges include consideration of inter-cellular variability in drug responses and integration of computational and experimental methods in safety pharmacology. Our aim is to evaluate the ability of *in silico* drug trials in populations of human action potential (AP) models to predict clinical risk of drug-induced arrhythmias based on ion channel information, and to compare simulation results against experimental assays commonly used for drug testing. A control population of 1,213 human ventricular AP models in agreement with experimental recordings was constructed. *In silico* drug trials were performed for 62 reference compounds at multiple concentrations, using pore-block drug models (IC_50_/Hill coefficient). Drug-induced changes in AP biomarkers were quantified, together with occurrence of repolarization/depolarization abnormalities. Simulation results were used to predict clinical risk based on reports of Torsade de Pointes arrhythmias, and further evaluated in a subset of compounds through comparison with electrocardiograms from rabbit wedge preparations and Ca^2+^-transient recordings in human induced pluripotent stem cell-derived cardiomyocytes (hiPS-CMs). Drug-induced changes *in silico* vary in magnitude depending on the specific ionic profile of each model in the population, thus allowing to identify cell sub-populations at higher risk of developing abnormal AP phenotypes. Models with low repolarization reserve (increased Ca^2+^/late Na^+^ currents and Na^+^/Ca^2+^-exchanger, reduced Na^+^/K^+^-pump) are highly vulnerable to drug-induced repolarization abnormalities, while those with reduced inward current density (fast/late Na^+^ and Ca^2+^ currents) exhibit high susceptibility to depolarization abnormalities. Repolarization abnormalities *in silico* predict clinical risk for all compounds with 89% accuracy. Drug-induced changes in biomarkers are in overall agreement across different assays: *in silico* AP duration changes reflect the ones observed in rabbit QT interval and hiPS-CMs Ca^2+^-transient, and simulated upstroke velocity captures variations in rabbit QRS complex. Our results demonstrate that human *in silico* drug trials constitute a powerful methodology for prediction of clinical pro-arrhythmic cardiotoxicity, ready for integration in the existing drug safety assessment pipelines.

## Introduction

Cardiotoxicity is one of the main causes of withdrawal during drug development, and identifying at early stages drugs that may cause adverse effects in specific human sub-populations is still a major challenge (Stevens and Baker, 2009; Laverty et al., 2011). Adverse effects can potentially lead to lethal arrhythmias, and are therefore a major cause of concern.

Before clinical testing, drugs undergo a thorough pipeline of preclinical testing, including identification of drug effects on cardiac ion channels (and particularly hERG), as well as in a variety of animal experiments (Leishman et al., 2012; Vargas et al., 2015). Animal models are good for predicting QT interval prolongation (Vargas et al., 2015), and some of them, including rabbit wedge preparations, rabbit isolated hearts and the *in vivo* atrioventricular block dog, have shown sensitive predictions of drug-induced Torsade de Pointes (TdP) (Valentin et al., 2004; Liu et al., 2006; Sugiyama, 2008). However, most of these studies only consider a small set of drugs. In fact, independent assessment against a larger number of compound (in the order of magnitude of those tested *in silico* in this contribution) highlight a prediction accuracy of 75% (Lawrence et al., 2008).

More recently, *in silico* and *in vitro* tests are considered as potentially important human-based tools for safety pharmacology evaluation, through the use of computational multiscale human modeling and human stem cell-derived cardiomyocytes (Bass et al., 2015; Rodriguez et al., 2016). Their profile has also been raised by the Comprehensive *in vitro* Proarrhythmia Assay (CiPA) initiative promoted by the pharmaceutical industries, the United States Food and Drug Administration (FDA), the Health and Environmental Sciences Institute and the Cardiac Safety Research Consortium (Sager et al., 2014; Colatsky et al., 2016).

The widespread translation of *in silico* modeling from academia to industrial and regulatory settings requires increasing the credibility of the models, understanding of their predictive power through comparison with existing experimental methods, and facilitating their uptake through the provision of software that can reduce the technical barriers of *in silico* methods for non-specialist users.

The aim of this study is to evaluate the ability of *in silico* drug trials using human ventricular model populations to predict the risk of drug-induced adverse cardiac events, based on ion channel information, and to identify ionic profiles underlying a higher risk of repolarization abnormalities. *In silico* drug trials were run for a large set of reference compounds with cardiac effects, and simulation results were analyzed to extract several biomarkers of drug pro-arrhythmic cardiotoxicity and compared against clinical reports of TdP arrhythmias. Because *in silico* drug trials are likely to be embedded in existing safety pharmacology pipelines and thus combined with experimental methodologies, it is important to evaluate their consistency with experimental recordings. Therefore, the outputs of the *in silico* drug trials for a sub-set of 15 reference compounds with varied modes of action were compared against the well-established electrocardiogram (ECG) recordings from isolated rabbit wedge preparations Lu et al. (2016) as well as the more recently considered technique of Ca^2+^-transient (CT) recordings from human induced pluripotent stem cell-derived cardiomyocytes (hiPS-CMs) (Lu et al., 2015; Zeng et al., 2016), even if with still controversial advantages (Abi-Gerges et al., 2017).

## Materials and methods

### Control population of human ventricular action potential (AP) models

All the *in silico* drug trials presented in this study were performed in a population of 1,213 human ventricular control models, built using the O'Hara-Rudy dynamic (ORd) model (O'Hara et al., 2011) as baseline and the methodology described by Britton et al. (2013) and further discussed by Muszkiewicz et al. (2016). The ORd human ventricular AP model was chosen for this study because of: (i) the large number of human ventricular experimental data obtained from more than 140 hearts used in its construction and evaluation; (ii) its ability to reproduce and probe pro-arrhythmic mechanisms, including repolarization abnormalities and APD alternans, as shown in multiple studies and reviewed by Britton et al. (2017); (iii) its choice within the CiPA initiative (Sager et al., 2014; Colatsky et al., 2016).

Ionic conductances were sampled in the [0–200]% range of the baseline model values, to include both healthy and potentially abnormal ionic current profiles (with low/high ion channel densities corresponding to loss/gain-of-function of specific ionic channels due to e.g., genetic mutations), but still with a healthy-looking AP. These are important as they have been implicated in increased pro-arrhythmic risk (Sanguinetti and Tristani-Firouzi, 2006; Itoh et al., 2016; Wang et al., 2016). Nine ionic conductances were considered: fast and late Na^+^ current (G_Na_ and G_NaL_ respectively), transient outward K^+^ current (G_to_), rapid and slow delayed rectifier K^+^ current (G_Kr_ and G_Ks_), inward rectified K^+^ current (G_K1_), Na^+^-Ca^2+^ exchanger (G_NCX_), Na^+^-K^+^ pump (G_NaK_), and the L-type Ca^2+^ current (G_CaL_).

Only the AP models exhibiting a phenotype in agreement with human experimental data from undiseased hearts (Britton et al., 2017) were selected for the control population, which consists of 1,213 models. A more detailed description of the control population used in this study is included in the Supplementary Material, together with the experimental AP biomarker ranges used for the calibration process (Table S1) and the scaling factors of the ionic conductances for the 1,213 models (Table S2).

### *In silico* drug assay design

A total of 62 reference compounds were considered in this study. The list includes antiarrhythmic drugs in Classes I, III, and IV, as well as other drugs used for different purposes but with known effects on cardiac ion channels. Most of these drugs have a multichannel action, which makes prediction and interpretation of cardiotoxicity challenging. Drugs were selected to include all the ones in Kramer et al. (2013), as well as 15 compounds widely used as reference compounds, which were characterized in more depth both in simulations and experiments and are listed in Table 1.

**Table 1:** List of the 15 compounds considered for *in silico* drug assays comparison against *in vitro* hiPS-CMs and *ex vivo* rabbit wedge preparations, including a short description and the clinical TdP Risk category based on CredibleMeds® (Woosley and Romer, 1999).

**Compound**		**Description**	**TdP risk category**
1	BaCl_2_	Barium Salt	0
2	Bepridil	Antiarrhythmic Class IV	1
3	Dofetilide	Antiarrhythmic Class III	1
4	Flecainide	Antiarrhythmic Class Ic	1
5	Lidocaine	Antiarrhythmic Class Ib Local Anaesthetic	0
6	Mexiletine	Antiarrhythmic Class Ib	0
7	Moxifloxacin	Antibiotic	1
8	Nimodipine	Used for Hypertension	0
9	Nisoldipine	Used for Hypertension	0
10	Phenytoin	Antiarrhythmic Class Ib Antiepileptic	0
11	Primidone	Anticonvulsant	0
12	Procainamide	Antiarrhythmic Class Ia	1
13	Ranolazine	Antianginal	3
14	Sparfloxacin	Antibiotic	1
15	Verapamil	Antiarrhythmic Class IV	0

*Risk categories from CredibleMeds® (Woosley and Romer, 1999): 1, high TdP risk; 3, conditional TdP risk; 0, no TdP risk (drugs not included in the CredibleMeds® database)*.

Each drug was assigned to a TdP risk category, based on the classification by CredibleMeds® (Woosley and Romer, 1999), available on www.crediblemeds.org (as of July 2017): 1 (high risk), the drug prolongs the QT interval and is clearly associated with a known TdP risk, even when taken as recommended; 2 (possible risk), the drug prolongs the QT interval, but there is a lack of evidence of TdP risk when taken as recommended; 3 (conditional risk), the drug is associated with TdP but only under certain circumstances, e.g., excessive dose or interaction with other drugs; NC (not classified), the drug was reviewed by CredibleMeds® but the evidence available was not enough to assign it to any of the previous categories, and therefore no action was taken. Of the 62 compounds, 24 are classified as high risk and 13 as potential/conditional risk, for a total of 37 drugs associated with TdP risk. Verapamil (classified as NC) and the remaining 24 compounds (not listed) are considered as TdP category 0 (no TdP risk) for the purpose of this study.

Drug effects were simulated using a simple pore-block model consistent with data available for drug/ion channel interactions, consisting of IC_50_ and Hill coefficient (h) for each drug/ion channel. Up to 7 ion channels were considered for this study: fast Na^+^ current (I_Na_), rapid/slow delayed rectified K^+^ current (I_Kr_/I_Ks_), transient outward K^+^ current (I_to_), L-type Ca^2+^ current (I_CaL_), inward rectifier K^+^ current (I_K1_), and late Na^+^ current (I_NaL_). The experimental IC_50_ and h used for the drug assays were collected mainly from three different sources: (i) our internal database, measured with either manual or automated patch-clamp techniques (when the IC_50_ concentration was not reached in the experiments, an estimate was computed from the percentage of block at the maximum tested concentration, with h equal to 1); (ii) (Kramer et al., 2013), data acquired with automated patch-clamp; (iii) (Crumb et al., 2016), data acquired with manual patch-clamp.

For the compounds that were included in more than one of these datasets, multiple inhibitory profiles were considered to investigate the impact of variability in drug characterization. Each IC_50_ and h set was simulated separately, resulting in 87 different drug trials: each trial is referred to with the name of the compound together with a roman numeral, to differentiate multiple entries (e.g., Bepridil I, Bepridil II, Bepridil III).

Multiple concentrations were investigated for each compound, chosen to match those used in the experimental drug assays, as well as to explore different multiples of the maximal effective free therapeutic concentration (EFTPC_max_), up to 100-fold. The EFTPC_max_ values were taken from literature, mainly from Kramer et al. (2013) or Crumb et al. (2016). When multiple values were found for the same compound, the higher one was considered for simulations.

The full list of compounds, together with the IC_50_, Hill coefficient and the EFTPC_max_ used for *in silico* drug trials is provided in Table S3.

### Simulations and simulated data analysis

All the simulations presented in this study were conducted using Virtual Assay (v.1.3.640 2014 Oxford University Innovation Ltd. Oxford, UK), a user-friendly C^++^ based software package with a graphical user interface for *in silico* drug assays, to facilitate its use by non-experts in computational modeling, and available upon request. Virtual Assay uses the ordinary differential equation solver CVODE, part of the open-source Sundials suite (Hindmarsh et al., 2005; Serban and Hindmarsh, 2005), implementing time adaptive Backward Differentiation Formulas with relative and absolute tolerance equal to 1e-5 and 1e-7, respectively. Our results could therefore be replicated using other software products, as Matlab (Mathworks Inc. Natwick, MA, USA) or Chaste (Pitt-Francis et al., 2008). As an example, a comparison of simulations obtained with Virtual Assay and with the Matlab solver ode15s (Shampine and Reichelt, 1997) is shown in the Supplemental Material, Figure S2. Simulation results were analyzed both in Virtual Assay and in Matlab.

Following drug application, all models were stimulated at 1 Hz for 150 beats, and the last AP trace in each simulation was analyzed. AP biomarkers were extracted, including: AP duration at 40, 50, and 90% of repolarization (APD_40_, APD_50_, APD_90_); APD_90_ dispersion, defined as the difference between the maximum and minimum value of APD_90_ in the population (ΔAPD_90_), AP triangulation, defined as the difference between APD_90_ and APD_40_ (Tri_90−40_); maximum upstroke velocity (dV/dt_MAX_); peak voltage (V_peak_); resting membrane potential (RMP), computed as in Britton et al. (2017). Drug-induced changes in those biomarkers are presented as percentage change in median with drug, compared to control (no drug).

All AP traces were automatically checked for repolarization and depolarization abnormalities (RA and DA, respectively). RA were defined as the presence of a positive derivative of the membrane potential (V_m_) 150 ms after the AP peak (representative of early after-depolarizations, EADs), or when the membrane potential did not reach the resting condition following an AP upstroke (V_m_ > −40 mV) by the end of the beat. DA were defined as AP traces in which the upstroke phase was compromised, i.e., when the max upstroke V_m_ was lower than 0 mV, or when the time needed to reach 0 mV was longer than 100 ms.

Drugs were classified as risky when RA occurred in the population of models at different concentrations, based on: true positives (drug with reports of TdP risk classified as risky); true negatives (drug with no reports of TdP risk classified as safe), false positives (drug with no reports of TdP risk, classified as risky); false negatives (drugs with reports of TdP risk, classified as safe). The performances of the classification were evaluated based on: sensitivity, defined as the number of true positives divided by the sum of true positives and false negatives; specificity, defined the number of true negatives divided by the sum of the true negatives and false positives; accuracy, defined as the sum of true positives and true negatives divided by the total number of drugs. Classification results based on RA were compared against the ones obtained for APD prolongation at 10x EFTPCmax. APD_90_ prolongation threshold to define risk was fixed to 6%, considering the correspondence between QTc and APD_90_, and the current guidelines suggesting QTc prolongation >20 ms (which correspond to 5.7% for a normal QT of 350 ms) as a definite risk factor for TdP (Salvi et al., 2010). Results for the population of models were also compared against the same results obtained with the single ORd model.

A scoring system was developed by integrating RA occurrence at multiple concentrations. The fraction of models developing RA was multiplied by a factor inversely related to the drug concentration at which those RA occur (e.g., 1/100 for RA occurring at 100x EFTPC_max_). Contributions from all the different concentrations were added together, and the total score was normalized, according to the following formula (where *nRA*_*i*_ is the number of models showing RA at the tested concentration *i*, w_i_ = EFTPC_max_ / *i* is the weight inversely related to the tested concentration *i*, and n_mod_ is the total number of models in the population).

(1) $$TdP\;score=\frac{\sum_{i}\left(w_{i}\text{*}nRA_{i}\right)}{n_{mod}\text{*}\sum_{i}\left(w_{i}\right)}$$

The TdP score thus obtained varies between 0 and 1, where 0 corresponds to a drug with no RA, and 1 to a drug which develops 100% of RA at every concentration. By using the proposed score, RA are considered more severe when occurring at low concentrations and/or affecting a high fraction of the population of models.

### Experimental drug assays

*In silico* results were compared with properties computed from ECGs in rabbit wedge preparations and CT recordings from hiPS-CMs. Experimental data were acquired for the 15 compounds listed in Table 1 at multiple concentrations, as described below.

Recordings of ECGs from left ventricular rabbit wedge preparations have been previously described and partly published in Lu et al. (2016). The biomarkers extracted include QRS complex and QT interval duration, defined as the time from the onset of the QRS complex to the point at which the final downslope of the T wave crossed the isoelectric line.

CT recordings from hiPS-CMs (Cor 4U) were acquired as part of this study on pre-plated preparations from Axiogenesis (Cologne, Germany). Full method details are included in the Supplementary Material. Quantified biomarkers included CT beat rate (CTBR) and duration at 90% of the initial base value (CTD_90_), known to be correlated with APD (Gauthier et al., 2012; Spencer et al., 2014), similarly to other studies (Lu et al., 2015; Zeng et al., 2016).

All experimental results are presented as median percentage changes with respect to the baseline. Drug-induced changes in experimental values need to be compared against the effect measured without drugs, i.e., with vehicle, defining cut-off values. In the rabbit wedge, the changes measured with the vehicle were always quite small: <5% for QT and <3% for QRS. On the other hand, in CT assays using hiPS-CMs, the lower and upper limit were 19% and 24% of the baseline, with 95% confidence interval (*n* = 222 vehicle controls). Therefore, only CTD_90_ prolongations >25% were considered relevant for this assay. *In silico*, no biomarker changes are observed when the drug effect is not included, since the models are paced until steady state: therefore, the cut-off value is equal to 0%. Statistical analysis was performed with the Wilcoxon-Mann-Whitney Test by using R Project for Statistical Computing, and *p* < 0.05 was considered as significant. Very small *p*-values (e.g., *p* < 1e-6) were obtained in all simulation results due to the high number of models considered, and therefore we focus on the magnitude of the differences observed (White et al., 2014).

## Results

### *In silico* drug assays: drug-induced changes in action potential (AP) biomarkers

*In silico* drug trials for a total of 62 reference compounds were performed in the control population of 1,213 human ventricular AP control models, based on the ORd model (O'Hara et al., 2011) and constructed as described in Methods. Before drug application, all the models exhibit a healthy-looking AP phenotype, in agreement with human experimental recordings from un-diseased hearts (Figure S1A). When including drug effect for concentration up to 100-fold the EFTPC_max_, each model responds in a different way, depending on its underlying ionic properties. We first evaluated drug-induced changes in the AP biomarkers.

Figure 1 shows APD_90_ and dV/dt_MAX_ distributions from the *in silico* population for 5 compounds (Dofetilide I, Flecainide I, Nimodipine, Ranolazine I, and Verapamil II) at multiple concentrations. Extended results for additional compounds, including all AP biomarkers, are shown in Figures S3–S31.

**Figure 1:**
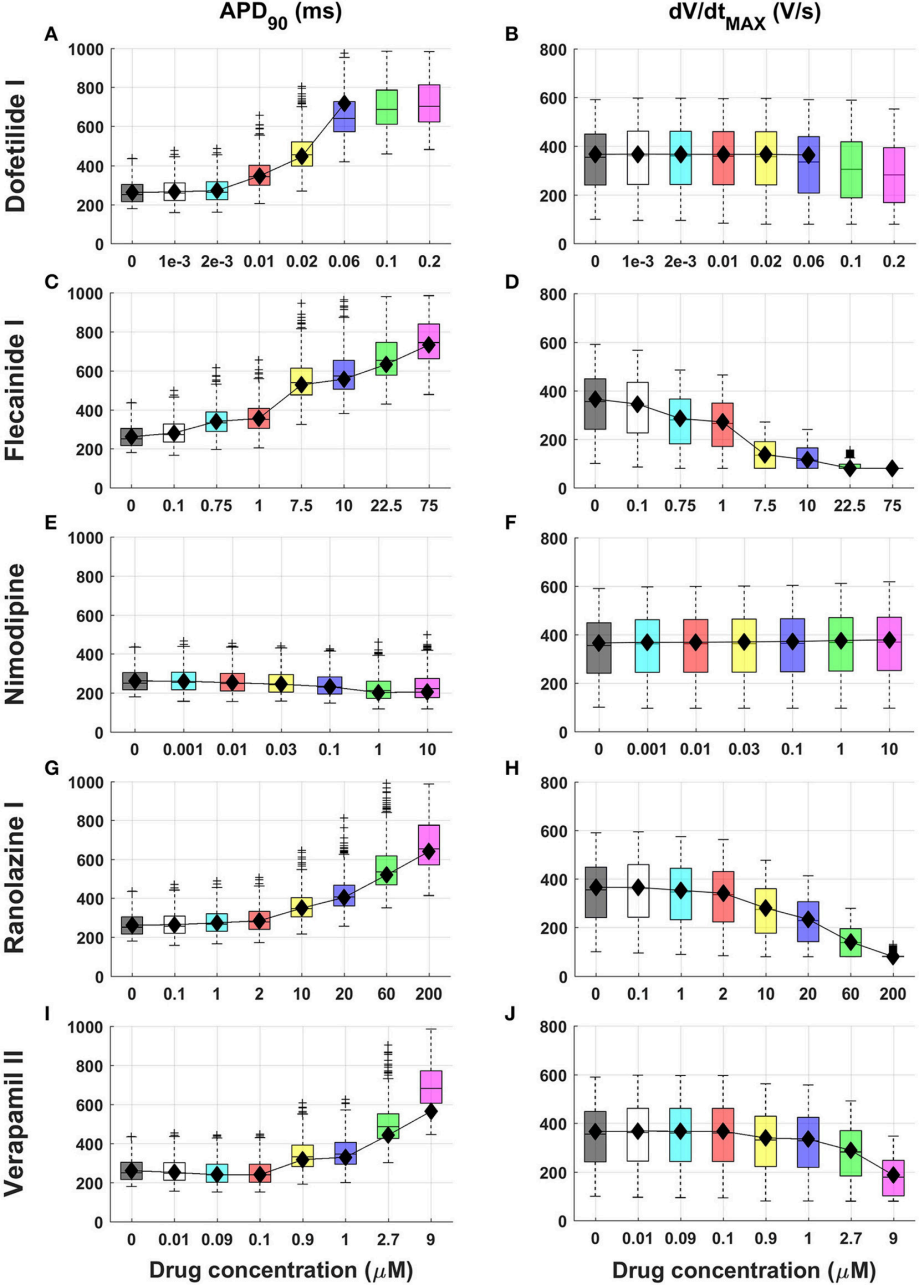
Explanatory examples of *in silico* drug trial results in a population of human computational models, showing drug-induced changes on APD_90_ and dV/dt_MAX_ (left and right column, respectively) for 5 compounds (Dofetilide I, Flecainide I, Nimodipine, Ranolazine I and Verapamil II). Results are presented as boxplots of AP biomarkers for the population of human ventricular models at increasing concentrations **(A–J)**. Results for the single ORd model are shown as black diamonds. On each box, the central mark is the median of the population, box limits are the 25 and 75th percentiles, and whiskers extend to the most extreme data points not considered outliers, plotted individually as separate crosses. Extended results for the selected 15 reference compounds, including all the AP biomarkers, are available in the Supplementary Material, Figures S3–S31.

Most drugs (all except Nimodipine and Nisoldipine) result in APD prolongation at 40, 50, and 90% of repolarization, as well as increased AP triangulation (Tri_90−40_), mainly as a result of hERG channel block (e.g., Figure 1, left column and A–C in Figures S3–S31). For 30x EFTPC_max_ dose, Flecainide III, Bepridil I, and Dofetilide III showed the largest APD_90_ prolongations (+180%, +175%, and +157%, respectively).

APD prolongation caused by BaCl_2_ (mainly due to I_K1_ block) is stronger in APD_90_ compared to APD_40_ and APD_50_ (Figures S3A–C). As a secondary effect of I_K1_ block, BaCl_2_ leads to a decrease in RMP (Figure S3H), which also exhibits larger variability, while for all the other compounds it remains almost constant (H in Figures S3–S31).

Consistent with their expected mode of action, class I anti-arrhythmic drugs (Procainamide, Lidocaine, Mexiletine, Phenytoin, Flecainide) as well as other drugs affecting Na^+^ channels as secondary effect (e.g., Bepridil) show a strong decrease of upstroke velocity (e.g., Figures 1D,H), together with a decrease of V_peak_ (F, G in Figures S3–S31).

Verapamil II represents an interesting example of the combined block of I_Kr_ and I_CaL_ (Figure 1I). For low concentrations (0.01–0.1 μM) the Ca^2+^ block is predominant, and APD_90_ is slightly decreased (−1 and −4% respectively), whereas I_Kr_ block compensates its effects for higher concentrations (>0.5 μM), resulting in a clear APD prolongation (e.g., +38% for 1 μM). Verapamil II also leads to slower AP upstroke for high concentrations (Figure 1J).

Results obtained using the baseline ORd model (Figure 1 and Figures S3–S31, black diamonds) are in overall agreement with the range of AP biomarkers in the population of human models. This is with the exception of cases in which the baseline ORd yields abnormal APs for high doses of certain drugs (e.g., Dofetilide I 0.1 and 0.2 μM, Figures 1A,B). In those cases, the human population of models still allows exploration of the full concentration range for each compound.

### *In silico* characterization of drug-induced phenotypic variability

**Figure 2:**
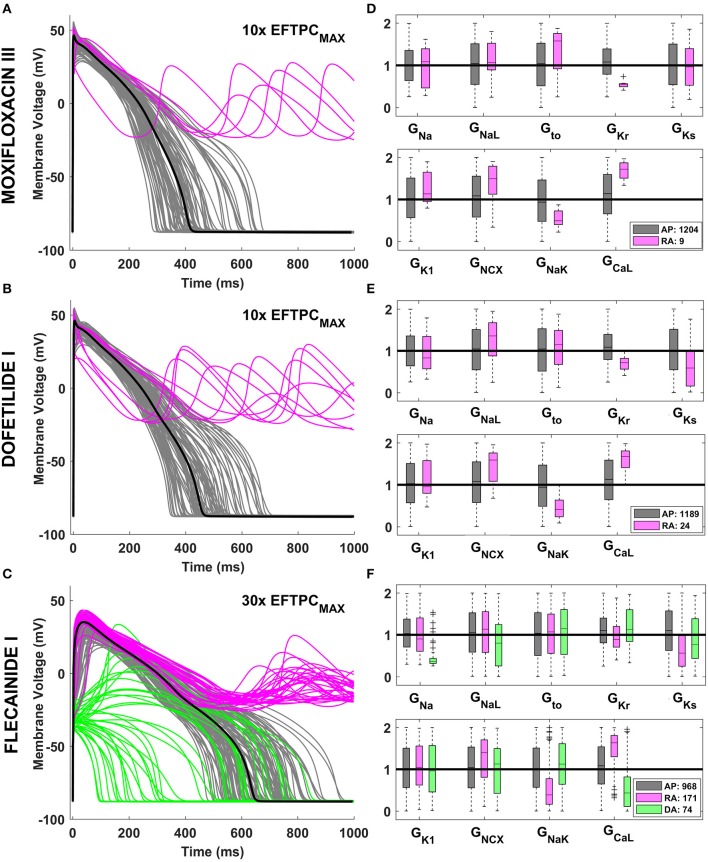
Explanatory examples of variability in drug response in the *in silico* population of human AP models, with the underlying ionic mechanisms. Representative AP traces of different drug-induced AP phenotypes are shown on the left side for Moxifloxacin III **(A)**, Dofetilide I **(B)**, and Flecainide I **(C)** at selected concentrations. Models with a normal AP are shown in gray, while models displaying RA and DA are shown in pink and green, respectively. In each panel, the baseline ORd model is highlighted in black. The distribution of ionic conductances for the different AP phenotypes is shown on the right side **(D–F)**, by using boxplots of the corresponding scaling factors, and with the same color code. For each conductance, the values shown (between 0 and 2) represent the scaling factors of the models in the population compared to the baseline ORd model, which had all the scaling factors equal to 1. Boxplots description as in Figure 1.

Drug action resulted in an increase in the phenotypic variability yielded by the human ventricular population, as illustrated in Figure 2 for Moxifloxacin III (Figure 2A) and Dofetilide I (Figure 2B), mainly blocking I_Kr_, and for Flecainide I (Figure 2C), inhibiting both I_Kr_ and I_Na_. Following drug application, some models in the *in silico* population display normal but prolonged APs (gray traces) while a fraction develop repolarization abnormalities (RA, pink traces). Due to its strong effect on I_Na_, Flecainide I (Figure 2C) also cause an overall reduction of dV/dt_MAX_, visible in the upstroke phase of the AP, and depolarization abnormalities (DA, green traces) in specific models.

Quantitative analysis of the underlying ionic mechanisms reveals consistency on the mechanisms underlying RA and DA across different drugs and concentrations. Models displaying RA are characterized by low G_Kr_, and G_NaK_, and high G_CaL_ and G_NCX_, i.e., a reduced repolarization reserve (Figures 2D–F, pink vs. gray boxplots). Low G_Ks_ also plays a role when a larger fraction of the population displays RA (Figures 2D,E). Models displaying DA are characterized mainly by low G_Na_/G_CaL_/G_NaL_, i.e. the net inward current in the initial phase of the AP is reduced (Figure 2F, green vs. gray boxplots).

### Repolarization abnormalities occurrence predicts TdP risk

We hypothesized that occurrence of RA following drug application in the human population would be predictive of *in vivo* TdP, given the potential mechanistic link between them (El-sherif et al., 1990; Dutta et al., 2016). *In silico* drug trial predictions were evaluated against clinical reports of TdP using the TdP risk categories provided by CredibleMeds® (Woosley and Romer, 1999) and further described in Methods. When multiple inhibitory profiles were simulated for the same compound, the worse scenario was considered, i.e., the higher occurrence of RA, and the larger APD_90_ prolongation.

**Figure 3:**
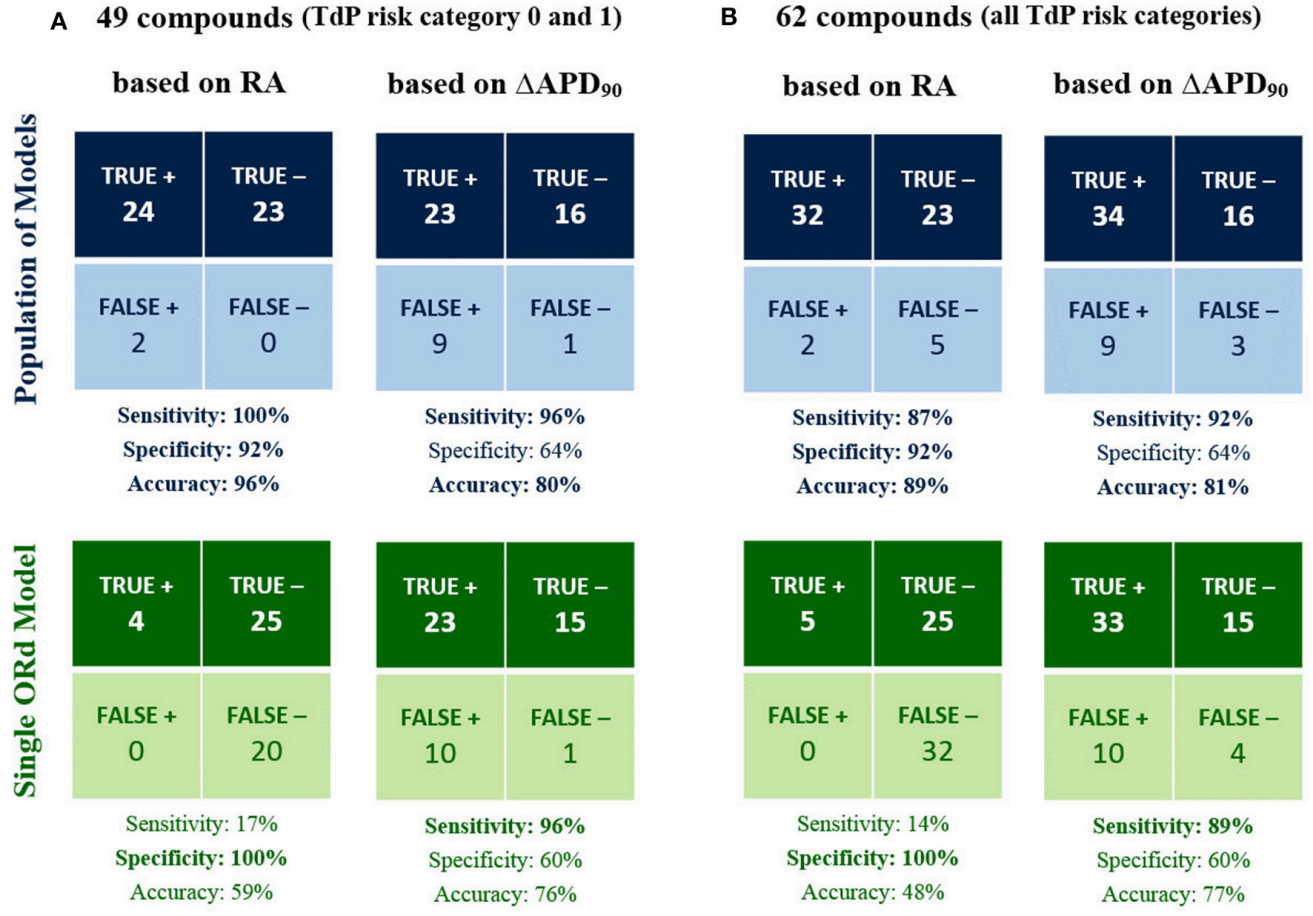
*In silico* prediction of *in vivo* TdP risk for the 49 compounds belonging to TdP risk category 0 and 1 **(A)** and for all the 62 tested compounds **(B)**. In each panel, predictions based on the occurrence of RA in any of the model at 1x, 10x, 30x, and 100x EFTPC_max_ (1st column) are compared against predictions based on APD_90_ prolongation >6% at 10x EFTPC_max_ (2nd column). Results obtained using the population of models (top half) are compared against the ones for the baseline ORd model (bottom half). High sensitivity/specificity/accuracy (>80%) are highlighted in bold.

Figure 3 shows the classification results for the 49 compounds with either high or no TdP risk (Figure 3A), and for the full set of 62 compounds (Figure 3B) based on RA occurrence up to 100x EFTPC_max_ and APD_90_ prolongation >6% at 10x EFTPC_max_ for both the population of models (top row), and the single ORd model (bottom row). Accuracy reached 96% for the classification of high vs. no TdP risk compounds using the RA-based classification with the *in silico* population of models, compared to 80% based on APD prolongation (Figure 3A). Using the single ORd model, the higher accuracy was 76% and was obtained using APD prolongation as biomarker.

When including also compounds with possible/conditional risk (Figure 3B), accuracy with the RA-based classification for the *in silico* population was 89%, compared to 81% based on APD prolongation (48 and 77% based on RA and APD prolongation, respectively, for the single ORd model).

In summary, all drugs with high TdP risk (category 1) were correctly identified as risky with the RA-based classification in the *in silico* population, and only 5 drugs with possible/conditional TdP risk (category 2 and 3) resulted as false negatives: Clozapine, Dasatinib, Paroxetine, Saquinavir, Voriconazole. It is worth noting that drugs with possible/conditional TdP risk lack evidence of pro-arrhythmic risk when taken as recommended: TdP reports are usually related to excessive dose or interaction with other drugs.

Overall, classification based on APD prolongation exhibited high sensitivity, but low specificity. Indeed, many compounds prolong APD without being associated with TdP risk, e.g., Verapamil, thus resulting in false positives. On the contrary, false positives are rare in the RA-based classification (Lidocaine and Mexiletine), and they only develop RA at the maximum tested concentration (100x EFTPC_max_). Indeed, both Lidocaine and Mexiletine are Class Ib anti-arrhythmic drugs, which have been associated with cardiotoxicity in case of overdose (Denaro and Benowitz, 1989).

RA-based classification is dependent on the maximum tested concentration: a higher concentration is more likely to provoke RA in the *in silico* population, thus increasing sensitivity but possibly decreasing specificity, since even safe drugs might lead to RA at very high doses. We reported here the classification results obtained for concentrations up to 100x EFTPC_max_, and a comparison between results for 30x and 100x EFTPC_max_ is included in the Supplementary Material (Figure S32).

### A new scoring system to evaluate *in vivo* risk of drug-induced TdP

**Figure 4:**
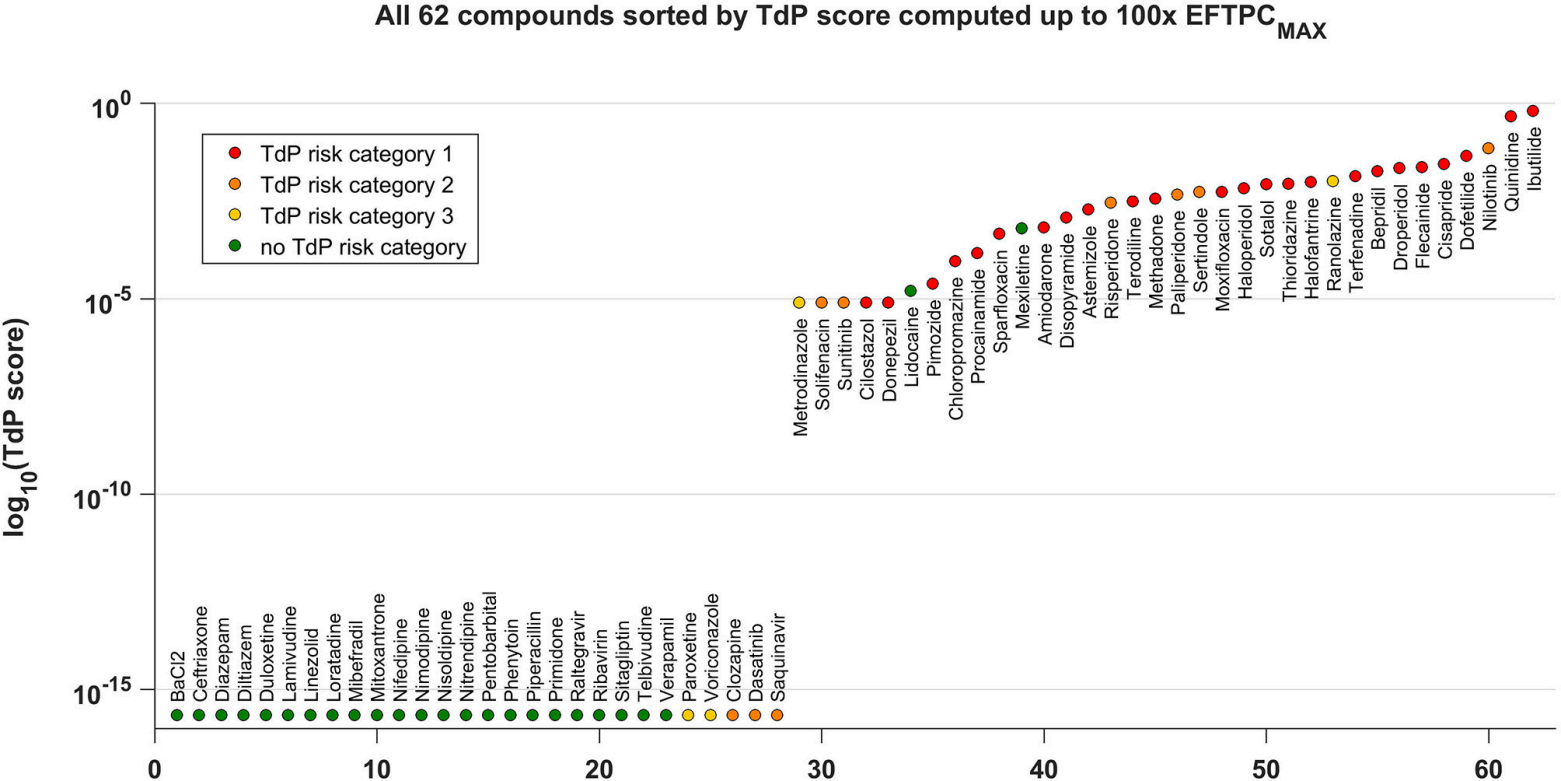
TdP score for all the 62 compounds, based on the occurrence of RA in the population of human AP models at 1x, 10x, 30x, and 100x EFTPC_max_. The TdP score, which varies between 0 and 1, was computed by taking into account both the fraction of models displaying RA and the concentrations at which RA occur, as described in Methods. The logarithmic scale was considered to maximize the visual separation between safe and risky drugs, and log_10_(0) was approximated with the machine precision (10^−16^). All compounds with no report of TdP risk (in green) have 0 as TdP score (left side), except for Lidocaine and Mexiletine. All high risk compounds (in red) have a high TdP score (right side). Most of the compounds with possible or conditional TdP risk (in orange and yellow, respectively) have a TdP score >0, except for Paroxetine, Voriconazole, Clozapine, Dasatinib, and Saquinavir.

Figure 4 shows all the tested compounds classified using the TdP score computed from the *in silico* drug trials using the fraction of models displaying RA at each tested concentration, as described in Methods. The TdP score varies from 0 to 1, and is higher when RA occur at low concentrations and/or affecting a high fraction of the population of models.

The distribution of compounds in the safe zone (TdP equal to 0, left side) and risky zone (TdP > 0, right side) reflects the classification results summarized in the confusion matrix with the higher (89%) accuracy (Figure 3). All safe compounds (no reported TdP risk, green dots) have a TdP score equal to 0, except Lidocaine and Mexiletine. All compounds with known risk of TdP (TdP risk category 1, red dots) have a positive score, and tend to be distributed toward the right end of the plot. Most of the compounds with possible or conditional TdP risk (TdP risk category 2 or 3, orange and yellow dots, respectively) have a positive score, except Clozapine, Dasatinib, Paroxetine, Saquinavir, Voriconazole. The TdP score is also dependent on the maximum considered concentrations. Again, higher concentrations lead to an increase in sensitivity while decreasing specificity. A comparison between the TdP scores computed up to 30x and 100x EFTPC_max_ is included in the Supplementary Material (Figure S33).

### *In silico* drug assays are in agreement with rabbit wedge and hiPs-CM experimental recordings

**Figure 5:**
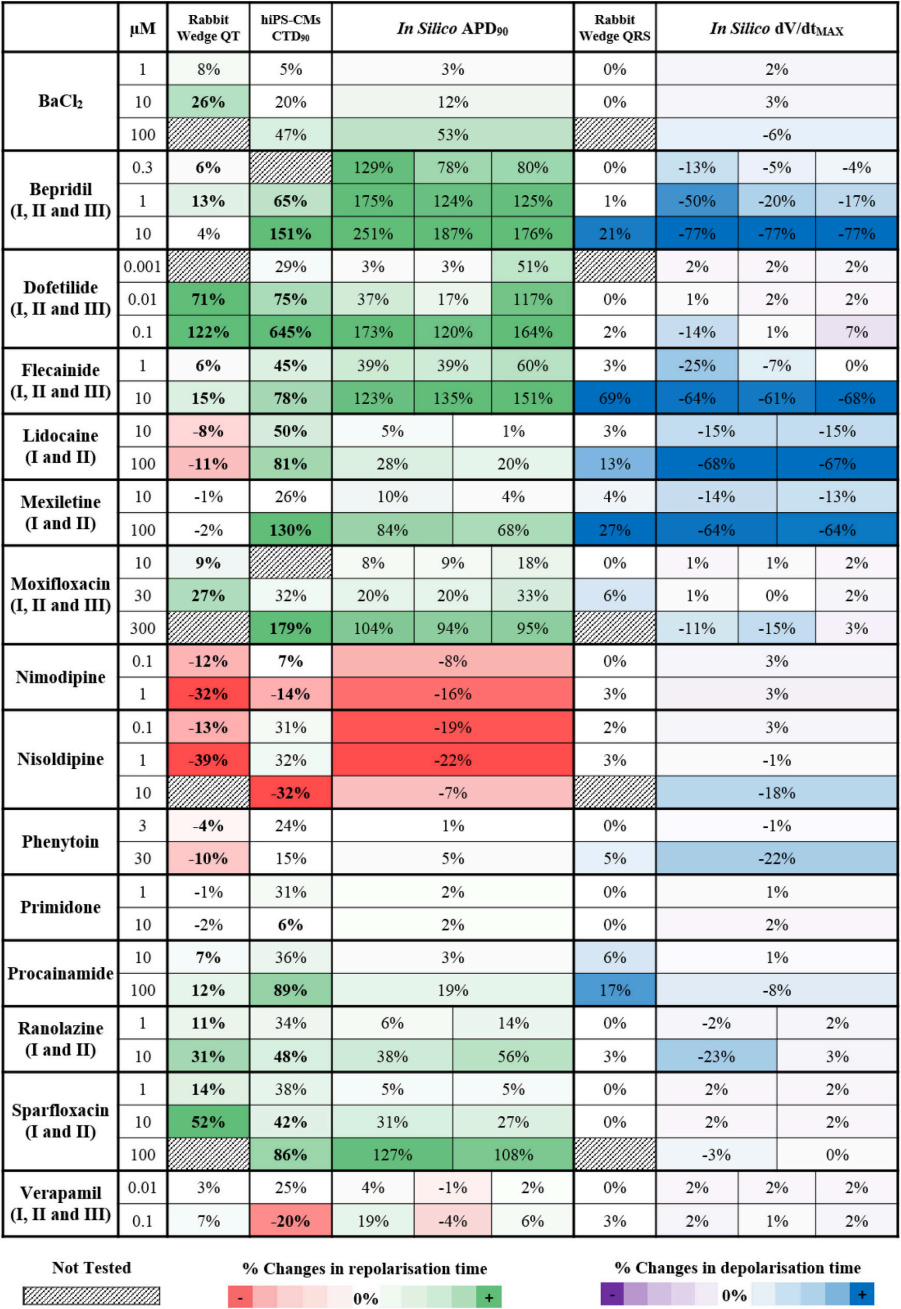
Qualitative and quantitative comparison of *in silico* drug trial results in the population of human ventricular AP models against ECG from rabbit wedge preparations and Ca^2+^ transient recordings from hiPS-CMs, for 15 reference compounds. On the left side (from red to green) are shown the drug-induced changes in the biomarkers related to the repolarization phase: QT interval in rabbit wedge, CTD_90_ in hiPS-CMs and APD_90_ *in silico*. On the right side (from purple to blue) are shown the drug-induced changes in the biomarkers related to the depolarization phase: QRS interval from rabbit wedge and dV/dt_MAX_ *in silico*. For each assay, colors are scaled to span from the 15th to the 85th percentiles of the % changes observed in the biomarkers when considering drug effects, compared to no drug, and with respect to the cut-off values (3 and 5% for rabbit wedge QRS and QT, 25% for hiPS-CMs CTD_90_, and 0% for *in silico* APD_90_ and dV/dt_MAX_). To facilitate comparison, negative variations in dV/dt_MAX_ were considered as positive changes in the depolarization time, and vice versa. When multiple combinations of IC_50_ and h were tested in simulation for the same compound, the corresponding *in silico* result sections consist of multiple sub-columns. Statistically significant changes in experiments have been highlighted in bold.

*In silico* drug trials are likely to be used as an additional tool for drug safety assessment in combination with experimental methods. It is therefore important to evaluate the consistency between *in silico* results and experimental data. Thus, simulation results for the 15 reference compounds with varied actions on ion channels (Table 1) were compared against recordings obtained using rabbit wedge and hiPS-CM preparations, as two techniques considered in safety pharmacology. In Figure 5, changes in QT interval duration in rabbit wedge, CTD_90_ in hiPS-CMs and *in silico* APD_90_ (from red to green, left side) were compared to evaluate drug-induced changes in repolarization. Changes in QRS complex duration in rabbit wedge and *in silico* dV/dt_MAX_ were quantified to evaluate drug effects on depolarization (Figure 5, from purple to blue, right side). Negative variations in dV/dt_MAX_ are considered as positive changes in depolarization time (opposite sign), to facilitate the comparison.

Drug-induced effects on biomarkers are in overall agreement for all three methodologies. Figure 5 presents consistent increase/decrease of QT, CTD_90_ and APD_90_, as well as consistency between positive changes in QRS and reduction of dV/dt_MAX_. Variations are generally larger in the *in silico* APD_90_ than in QT interval in rabbit wedge, indicating higher sensitivity of the *in silico* assay, and the wider range of ionic scenarios evaluated in the virtual population than in the limited number of experiments.

For Verapamil at 0.1 μM, small changes were observed in both QT interval in rabbit wedge and simulated APD, whereas CTD_90_ in hiPS-CMs was reduced. Such a reduction in CTD_90_ in the hiPS-CMs is also accompanied by a significant increase in beating rate (CTBR +61%), which does not occur *in silico* and in the rabbit wedge experiments as these are paced externally.

For Lidocaine and Mexiletine, their main effect is fast I_Na_ block, which results in a decreased dV/dt_MAX_ in the *in silico* models, and a wider QRS complex in the rabbit wedge ECG. Furthermore, both *in vitro* CTD_90_ and *in silico* APD_90_ are prolonged, whereas QT interval decreases slightly (Lidocaine) or remains unchanged (Mexiletine) in rabbit wedge, suggesting that *in vitro* CT and *in silico* AP are more prone to display prolongation for non-selective Class I anti-arrhythmic drugs. It is worth noticing that the AP prolongation *in silico* is reduced when inhibition of the I_NaL_ current is taken into account in the simulations, corresponding to Lidocaine II and Mexiletine II (APD_90_ +84% vs. +68%, Mexiletine I vs. Mexiletine II, 100 μM). The tendency for prolongation under Na^+^ block of *in silico* AP and hiPS-CMs CT is confirmed also for another class I anti-arrhythmic drug (Phenytoin), which causes negligible changes in both CTD_90_ and *in silico* APD_90_, and a decrease in QT interval in rabbit wedge.

Bepridil constitutes a good example of multichannel block, intended to block I_CaL_, but also affecting I_Kr_ and I_Na_. In the rabbit wedge, the QT interval is relatively prolonged at low concentrations (0.3–1 μM), compared to more selective Ca^2+^ blockers (e.g., Nimodipine and Nisoldipine), and it goes back to normal (+4%) at 10 μM. Both hiPS-CMs CT and *in silico* AP are prolonged in a dose-dependent manner, confirming once again the higher sensitive to I_Kr_ block of these techniques.

Interestingly, BaCl_2_ effects are of smaller magnitude in hiPS-CMs compared to rabbit wedge preparation and *in silico* models: relevant CTD_90_ prolongation was detected only at 100 μM (+47%), while up to 10 μM (more than 2-fold BaCl_2_ IC_50_ for I_*K*1_) the drug-induced effects on CT were negligible. This may be due to differences in I_*K*1_ expression between the cell types considered (Liang et al., 2013; Kim et al., 2015).

*In silico* results for compounds with multiple sets of IC_50_ and h are in overall agreement with each other, even if the magnitude of drug-induced changes may vary. As an example, the decrease in dV/dt_MAX_ for the three variations of Flecainide is almost the same at 10 μM (−64, −61, and −68%, respectively), while for lower concentrations the differences between the three inhibition profiles are more noticeable (e.g., −25, −7, and 0% at 1 μM, respectively).

## Discussion

*In silico* human electrophysiology drug trials using a population of human AP models were conducted for 62 compounds with varied electrophysiological profiles to evaluate their ability to predict clinical pro-arrhythmic risk and their consistency with electrophysiological recordings currently considered in safety assessment.

The main findings of this simulation study are:

1. RA occurrence in populations of human models proves to be more predictive of clinical TdP risk than APD prolongation and standard biomarkers obtained with the single ORd model. Accuracy of 96% and specificity of 92% was obtained in the classification of high risk vs. safe drugs, compared to 80 and 64%, based on APD prolongation. 100% sensitivity was achieved considering RA in the population compared to 17% with the single ORd model.
2. Human virtual cardiomyocytes exhibiting RA in *in silico* drug trials displayed low repolarization reserve, caused by low I_Kr_/I_Ks_/I_NaK_ and high I_CaL_/I_NCX_, which is consistent with the prevalence of cardiotoxicity in patients with disease conditions such as myocardial ischaemia and heart failure. Low depolarization reserve caused by weak I_Na_/I_NaL_/I_CaL_ was associated with DA under I_Na_ block.
3. A TdP risk score was developed to translate the high accuracy of RA abnormalities for risky drug classification into a non-binary system considering both data for multiple concentrations and the frequency of RA. This metric is higher when RA occur in a large fraction of the population of models, and at lower concentrations, thus informing on the likelihood of drug-induced adverse cardiac events in the population.
4. Drug-induced APD changes *in silico* are consistent with the ones measured in QT interval from rabbit wedge ECGs and CTD recorded from hiPS-CMs. Drugs affecting the depolarization phase provoke a decrease of dV/dt_MAX_ *in silico* and a widening of QRS interval rabbit wedge ECGs. To show a qualitative and quantitative agreement between *in silico* drug trial results and two experimental models commonly used in safety pharmacology, is fundamental to build confidence in the integration of computer models for cardiotoxicity assessment.

Our results support the potential of RA in the *in silico* human population as a good predictor of clinical TdP risk, with sensitivity, specificity and accuracy higher or comparable to the ones obtained through animal studies (Valentin et al., 2009). RA-based classification for all 62 compounds reached 89% of accuracy, compared to 75% obtained for 64 compounds in rabbit isolated Langendorff heart model (Lawrence et al., 2006; Valentin et al., 2009). The *in vivo* atrioventricular block dog model showed sensitive predictions of drug-induced TdP, but in a limited set of 13 compounds (Sugiyama, 2008). Animal studies accuracy is higher when predicting QT prolongation rather that *in vivo* TdP risk: 85 and 79% for 19 compounds based on QT prolongation in *in vivo* dog studies and hERG assays, respectively (Valentin et al., 2009; Wallis, 2010); 90% accuracy for 40 compounds based on non-rodent QT prolongation (Vargas et al., 2015). However, “it is generally known that the sensitivity and the specificity of the QT interval prolongation as a surrogate marker of TdP is rather poor: only in 46% of the cases the TQT study results were concordant with the TdP risk classification, and 89% of drugs prolonging QT interval in thorough QT studies were approved by the FDA” (Wiśniowska et al., in press). This is confirmed in our study by the fact that RA-based predictions in the population of human models have higher accuracy compared to the ones based on APD prolongation, due to a higher specificity (92 vs. 64% for 62 compounds).

Our methodology also offers mechanistic insights into sub-populations at higher risk, which is a key advantage with respect to previous *in silico* and *in vitro* studies (Kramer et al., 2013; Lancaster and Sobie, 2016). We identify human *in silico* cardiomyocytes with high propensity to develop RA as those with low I_NaK_/I_Ks_/I_Kr_ and high I_NCX_/I_CaL_. The ionic profile is consistent with ionic remodeling in cardiac specific diseases such as heart failure (Carmeliet, 1999; Coppini et al., 2013; Coronel et al., 2013), suggesting disease modeling as crucial when investigating cardiotoxicity in response to drug action (Walmsley et al., 2013; Gomez et al., 2014; Elshrif et al., 2015; Dutta et al., 2016; Passini et al., 2016).

The range of concentrations considered for drug trials plays an important role in risk prediction. Expanding the concentration up to 100x EFTPC_max_ allows to account for possible overdose, but most importantly for inter-subject variability in protein binding and metabolism, which can lead to important different in blood concentrations in patients taking the same drug dose, or even hormones which might change the effect of the drug on ion channels (Shuba et al., 2001). As an example, Amiodarone is a very controversial drug, considered safe by most clinicians but at the same time known to be associated with TdP risk (Jurado Román et al., 2012), and indeed belonging to TdP risk category 1. Amiodarone is almost completely bound to plasma proteins: reported values in literature range from 95.6% (Lalloz et al., 1984; Latini et al., 1984) to 99.98% (Veronese et al., 1988). In addition, absorption following oral administration is erratic and unpredictable (Latini et al., 1984). This can lead to EFTPC_max_ variation of more than 100-fold, with a big impact on drug-induced ion channels block.

An additional consideration about *in silico* trials concerns variability of recorded IC_50_ values. We show one possible way to consider this variability, by evaluating its implications in the *in silico* human population. In most cases, results obtained with different IC_50_ values were in overall agreement, thus building confidence in the answer provided. Should these results disagree, leading to contrasting scenarios, new ion channel recordings and experiments might be required for further drug characterization and refined *in silico* predictions. This may incorporate for example more detailed models of ion channel structure, based on the most recent crystallographic studies on human ion channels (Sun and MacKinnon, 2017; Wang and MacKinnon, 2017). Other *in silico* tools are also available at the ion channel level, to evaluate potential drug effects on the hERG channel, using ligand-based (Durdagi et al., 2011; Braga et al., 2015; Chemi et al., 2017) or receptor-based (Brindisi et al., 2014; Dempsey et al., 2014) approaches.

Indeed, *in silico* results are strictly dependent on the quality and consistency of the data used as inputs, which include ion channel assays costing time and money. *In silico* trials are a cheap complement to experimental methods following ion channel screening, which for some channels is already routine (hERG).

When fully integrated in the early stages of drug development, *in silico* methods provide predictions to partly replace animal experiments, thus reducing the corresponding costs. Therefore, *in silico* drug trials are likely to play soon a major role in drug development, identifying drug cardiotoxicity in the pre-clinical phase, thus improving the quality of new candidate drugs and reducing drug failure at later stages.

Our results are obtained using experimentally-calibrated population of human models for *in silico* drug trials. The wide range of conductances considered includes extreme up- or down-regulation of ion channels, which can be linked to specific mutations or diseased conditions known to be pro-arrhythmic (Sanguinetti and Tristani-Firouzi, 2006; Itoh et al., 2016; Wang et al., 2016). Previous studies have also considered aspects of population variability for gender and age, mostly by changing cell volume and area (rather than ionic conductances) using a commercial software (Polak et al., 2012). The same software was also recently used to investigate potential drug-induced arrhythmias for 12 drugs (Abbasi et al., 2017). In that study, variability was taken into account by using different AP models (Ten Tusscher, 2003; Ten Tusscher and Panfilov, 2006; O'Hara et al., 2011) and different cell types (endo-, epi-, and mid-myocardium). However, their results were presented only for a single model (mid-) since it was the one most prone to develop drug-induced APD prolongation and EADs.

Our results also demonstrate consistency between human-based *in silico* simulations and recordings obtained from experimental models traditionally used in safety pharmacology, including rabbit wedge ECGs (Lu et al., 2016) and hiPS-CMs CT recordings. Previous *in silico* studies have focused on predictions of QT prolongation in human (Mirams et al., 2014; Lancaster and Sobie, 2016) and animal models (Bottino et al., 2006; Davies et al., 2012; Beattie et al., 2013). Evaluating the *in silico* results against experimental data is important as *in silico* tools are likely to be used in combination with experimental recordings for validation and identification of potential unknown effects. Importantly, our results also identifies discrepancies between *in silico* results and experimental and clinical data, when considering compounds with strong multichannel action, leading to large AP prolongation *in silico* and only a moderate increase of rabbit wedge QT. The potential causes of such discrepancies include: (i) differences in the balance of inward/outward currents in human adult cardiomyocytes as represented *in silico* with respect to rabbit wedge preparations, which may lead to higher sensitivity to hERG block. Indeed, it has been shown that APD prolongation due to I_Kr_ block is more pronounced in human, compared to rabbit (Bányász et al., 2011; O'Hara and Rudy, 2012); (ii) the fact that *in silico* results in our study are focused on single cell electrophysiology, as opposed to tissue (rabbit wedge) or whole heart, where coupling and other mechanisms may act to modulate AP duration, as supported by the fact that both *in silico* APD and hiPS-CMs CTD show larger prolongation compared to rabbit QT; (iii) the IC_50_ values were often estimated based on current blocks measured at low drug concentrations, while simulations explored much higher ones. This can therefore lead to an overestimation of current blocks, producing a larger AP increase than expected. Further work could address these important factors.

To conclude, this study demonstrates that *in silico* drug trials in populations of human cardiomyocyte models constitute a powerful methodology to predict clinical risk of arrhythmias based on ion channel information. This study also highlights ionic profiles that have a higher risk of developing drug-induced abnormalities. This methodology is therefore ready for its integration into the existing pipeline for drug cardiotoxicity assessment, and contribute to the reduction of animal experiments in the near future.

## Author contributions

All the authors conceived and designed the study; EP performed the *in silico* drug assays, analyzed the data, prepared the figures and drafted the manuscript; HL, JR, and AH performed experimental drug assays; RG, OB, and EP developed the software; EP, OB, AB, and BR interpreted the results; all the authors edited and revised the manuscript.

### Conflict of interest statement

HL, JR, AH, and DG are employees of Janssen Pharmaceutica NV. RG is an employee of Oxford Computer Consultants, Oxford, UK. The other authors declare that the research was conducted in the absence of any commercial or financial relationships that could be construed as a potential conflict of interest.

## Abbreviations

AP: Action potential
APD_XX_: Action potential duration at XX% of repolarization
CiPA: Comprehensive *in vitro* Proarrhythmia Assay
CT: Ca^2+^-transient
CTBR: Ca^2+^-transient beat rate
CTD_XX_: Ca^2+^-transient duration at XX% of the initial base value
DA: Depolarization abnormalities
dV/dt_MAX_: Maximum upstroke velocity
EADs: Early after-depolarizations
EFTPC_max_: Maximal effective therapeutic free concentration
G_X_, I_X_: conductance
h: Hill coefficient
hiPS-CMs: Human induced pluripotent stem cell-derived cardiomyocytes
IC_50_: Concentration for 50% channel inhibition
I_CaL_: L-type Ca^2+^ current
I_K1_: Inward rectifier K^+^ current
I_Kr_: Rapid delayed rectifier K^+^ current
I_Ks_: Slow delayed rectifier K^+^ current
I_Na_: Fast Na^+^ current
I_NaK_: Na^+^-K^+^ pump current
I_NaL_: Late Na^+^ current
I_NCX_: Na^+^-Ca^2+^ exchanger current
I_to_: Transient outward K^+^ current
ORd: O'Hara-Rudy dynamic human ventricular model
RA: Repolarization abnormalities
RMP: Resting membrane potential
TdP: Torsade de Pointes
Tri_90−40_: AP triangulation
V_m_: Membrane potential
V_peak_: Peak voltage.
