# Supplementary material for: Human In Silico Drug Trials Demonstrate Higher Accuracy than Animal Models in Predicting Clinical Pro-Arrhythmic Cardiotoxicity
Source: Front Physiol. 2017 Sep 12;8:668. doi: 10.3389/fphys.2017.00668 (PMC5601077; doi:10.3389/fphys.2017.00668)
Supplement: Supplementary file 1 [file DataSheet1.docx]

**Supplementary Material**

## Expanded Methods for human *in silico* drug trials

A population of 1,213 human ventricular action potential (AP) models was constructed in range with experimental human electrophysiological recordings (Britton et al., 2017; O’Hara et al., 2011).

An initial population of 3,000 models was generated based on the O’Hara-Rudy dynamic (ORd) human ventricular AP model (O’Hara et al., 2011), using the methodology previously described by (Britton et al., 2013). All models in the population share the same equations, but the maximum conductances of the 9 ionic currents most important to depolarisation and repolarisation properties of the human ventricular AP were sampled between 0% and 200% of their baseline value using Latin Hypercube sampling, to account for inter and intra-subject variability in ionic profiles in human cardiomyocytes. These include fast and late Na^+^ current (G_Na_ and G_NaL_, respectively), transient outward K^+^ current (G_to_), rapid and slow delayed rectifier K^+^ current (G_Kr_ and G_Ks_), inward rectified K^+^ current (G_K1_), Na^+^-Ca^2+^ exchanger (G_NCX_), Na^+^-K^+^ pump (G_NaK_) and the L-type Ca^2+^ current (G_CaL_). The [0-200]% sampling range was chosen to include both healthy and potentially abnormal ionic current profiles (with low/high ion channel densities corresponding to loss/gain-of-function of specific ionic channels due to e.g. genetic mutations), but still displaying a healthy-looking AP.

The initial population was paced at 1 Hz for 500 beats, to allow the human models to reach a steady state in which the intracellular concentrations of Na^+^ and K^+^ were stable. The last AP trace for each model was used to compute a set of AP biomarkers: AP duration at 40%, 50% and 90% of repolarisation (APD_40,_ APD_50,_ APD_90_); APD_90_ dispersion, defined as the difference between the maximum and minimum value of APD_90_ in the population (ΔAPD_90_), AP triangulation, defined as the difference between APD_90_ and APD_40_ (Tri_90-40_); maximum upstroke velocity (dV/dt_MAX_); peak voltage (V_peak_); resting membrane potential (RMP). All AP biomarkers were computed as in (Britton et al., 2017).

The population was then filtered based on the extensive dataset used to build the ORd model itself (Britton et al., 2017; O’Hara et al., 2011). Table S1 shows the 7 AP biomarkers used for the calibration process and their admissible ranges, defined by considering the minimum and maximum experimental values. V_peak_ upper limit was extended from 40 to 55 mV, since AP amplitude is lower in tissue compared to single cell (Clayton et al., 2011); indeed, the original ORd model itself has a basal amplitude of 45 mV. Only the models with all AP biomarker values within the experimental ranges were included in the final control population used for *in silico* drug assays (1,213 in total). The ionic conductance values for these 1,213 models are included in Table S2.

Figure S1A illustrates the AP properties of the 1,213 human ventricular models in the population, in agreement with experimental ranges. Differences in AP duration and morphology depend on the specific ionic profile of each model. Figure S1B illustrates the distribution of the 9 ionic conductances in the experimentally-calibrated population. Models with G_Na_ or G_Kr_ smaller than ~30% of the original model value were rejected from the calibrated population as they yielded APs with biomarkers outside experimental ranges. Figure S1C shows the agreement between the ranges of AP biomarkers in the control population and experimental recordings in human preparations.

The range of ionic profiles in the calibrated human *in silico* population is meant to be wide, as this enables exploring a wide range of scenarios to identify potential arrhythmic risk due to drug effects. Please note that this is not meant to be a parameter estimation exercise to identify the distribution of ionic currents in the specific preparations used in the experiments, for at least two reasons: (1) this would require additional constrains to be imposed during calibration, as discussed in the original paper by (Britton et al., 2013); (2) the specific ionic profile of cardiomyocytes in experimental preparations is constantly changing, due to a variety of factors such as changes in the preparation itself, hormones, circadian rhythm and temperature, amongst other factors, particularly *in vivo*, which is the focus of our investigations. This important points as well as others have been discussed at length in previous publications (Britton et al., 2013, 2017; Gemmell et al., 2014; Liberos et al., 2016; Muszkiewicz et al., 2016; Passini et al., 2016; Reilly et al., 2016; Sanchez et al., 2014; Zhou et al., 2016).

In this study, all the simulations were conducted using Virtual Assay (v.1.3.640 © 2014 Oxford University Innovation Ltd. Oxford, UK), as described in the main manuscript. In Virtual Assay, the simulation of the initial population of 3,000 models (paced for 500 beats each) requires about 3 hours in an Intel® Core™ i7-4770 CPU @ 3.40 GHz, 16GB RAM with Windows 7 Enterprise 64-bit. Drug trials for one compound require about 45 minutes for each concentration, in the calibrated population of 1,213 models, stimulated for 150 beats at 1Hz, and using the same Desktop Computer. A comparison of simulations obtained with Virtual Assay and with the Matlab solver ode15s (Shampine and Reichelt, 1997) is shown in Figure S2.

Drug effects were simulated using a simple pore-block model (Brennan et al., 2009), i.e. by decreasing the specific ion channel conductances, based on an experimental IC_50_ and Hill coefficient of the drug, and its concentration. The choice of the simple model was consistent with the information available on the drug effects on ion channel for the 62 compounds. A similar analysis could be conducted using state-dependent model of drug/ion channel interactions should the required information become available. This would however be expensive and time consuming for such a large number of compounds.

## Detailed methods for CT recordings from hiPS-CMs

Living, pre-plated hiPS-CMs (Cor.4U^®^ cardiomyocytes) were obtained from Axiogenesis (Cologne, Germany). Cells were seeded in fibronectin-coated 96-well plates at a density suited to form a monolayer and maintained in culture in a stage incubator (37^o^C, 5% CO_2_). The experiments with test drugs were carried out 1 to 5 days after cell delivery (5-9 days after plating). The spontaneous electrical activity was recorded as the calcium dye-fluorescence signal integrated over the whole well, using Calcium 5 Kit (Molecular Devices). Fluorescence signals (CT morphology) were measured using Functional Drug Screen System (FDSS 6000; modified system; Hamamatsu, Japan) and the records subsequently analyzed off-line, using Notocord-Hem software (version 4.3).

The following parameters of the CT morphology were measured:

- CT beat rate (CTBR)
- CT amplitude (CTamp)
- CTD_90_: Ca^2+^ transient duration at 90% (time to 90% of the initial base value)

If *‘arrhythmia-like’* activity such as ‘*early afterdepolarization-like’* (EAD-like) events, ‘*ventricular tachycardia/ventricular* *fibrillation-like’* (VT/VF-like) events, or ‘*stop beating*’ of the cells (no CT) are noticed during the experimental period they are reported (visual check).

The test compounds were dissolved in DMSO to obtain a stock solution of 1000x the highest test concentration. Further dilutions were made with DMSO to obtain concentrations of 1000x intended concentration. On the experiment day these solutions are diluted with Tyrode (Sigma), supplemented with 10 mM HEPES to solutions containing twice the intended concentration (compound plate: 2x intended concentration). Final DMSO concentration in test solutions and vehicle control was 0.1%.

Before starting the experiment, a test run was done. The Ca^2+^ transients were measured for 4 minutes to check synchronous beating of the cardiomyocytes in each well. All 96 wells were measured simultaneously (sampling interval: 0.06 s, short exposure time: 10 ms; excitation wavelength 480 nm; emission wavelength 540 nm; The FDSS warmed to 37 ^o^C).

If all or most wells show synchronous beating the 96 well plate was measured again 3 times:

- t=0: control period (1 min) + compound addition, followed for 3 min
- t=10: measured from 9 to 13 min after compound addition
- t=25: measured from 24 to 28 min after compound addition

The CT biomarkers considered in this study are the ones measured for t=25.

**Supplementary Tables and Figures**

**Table S1:** Admissible ranges for the 7 AP biomarkers used to calibrate the initial population of human ventricular AP models in Virtual Assay, based on human AP recordings (Britton et al., 2017; O’Hara et al., 2011).

| **AP Biomarker:** | **Min Value** | **Max Value** |
| --- | --- | --- |
| APD_40_ | 85 ms | 320 ms |
| APD_50_ | 110 ms | 350 ms |
| APD_90_ | 180 ms | 440 ms |
| Tri_90-40_ | 50 ms | 150 ms |
| dV/dt_MAX_ | 100 V/s | 1000 V/s |
| V_peak_ | 10 mV | 55 mV |
| RMP | -95 mV | -80 mV |

**APD_X_**: AP duration at X% of repolarisation; **Tri_90-40_**: AP triangulation, defined as the difference between APD_90_ and APD_40_; **V_peak_**: peak voltage; **RMP**: resting membrane potential; **dV/dt_MAX_**: maximum upstroke velocity.

**Table S2**. Ionic conductances for the control population of human ventricular models. The values shown (between 0 and 2) represent the scaling factors of the 1,213 models in the population compared to the baseline ORd model (with all scaling factors equal to 1).

*Table S2 is provided as a separate Excel file, including also References and Notes.*

**Table S3**. IC_50_ and Hill coefficient (h) values used as inputs for the 87 *in silico* drug trials. For each compound, the EFTPC_max_ and TdP risk category are also included. Data come from different sources, and all the references are listed in the table.

*Table S3 is provided as a separate Excel file, including also References and Notes.*


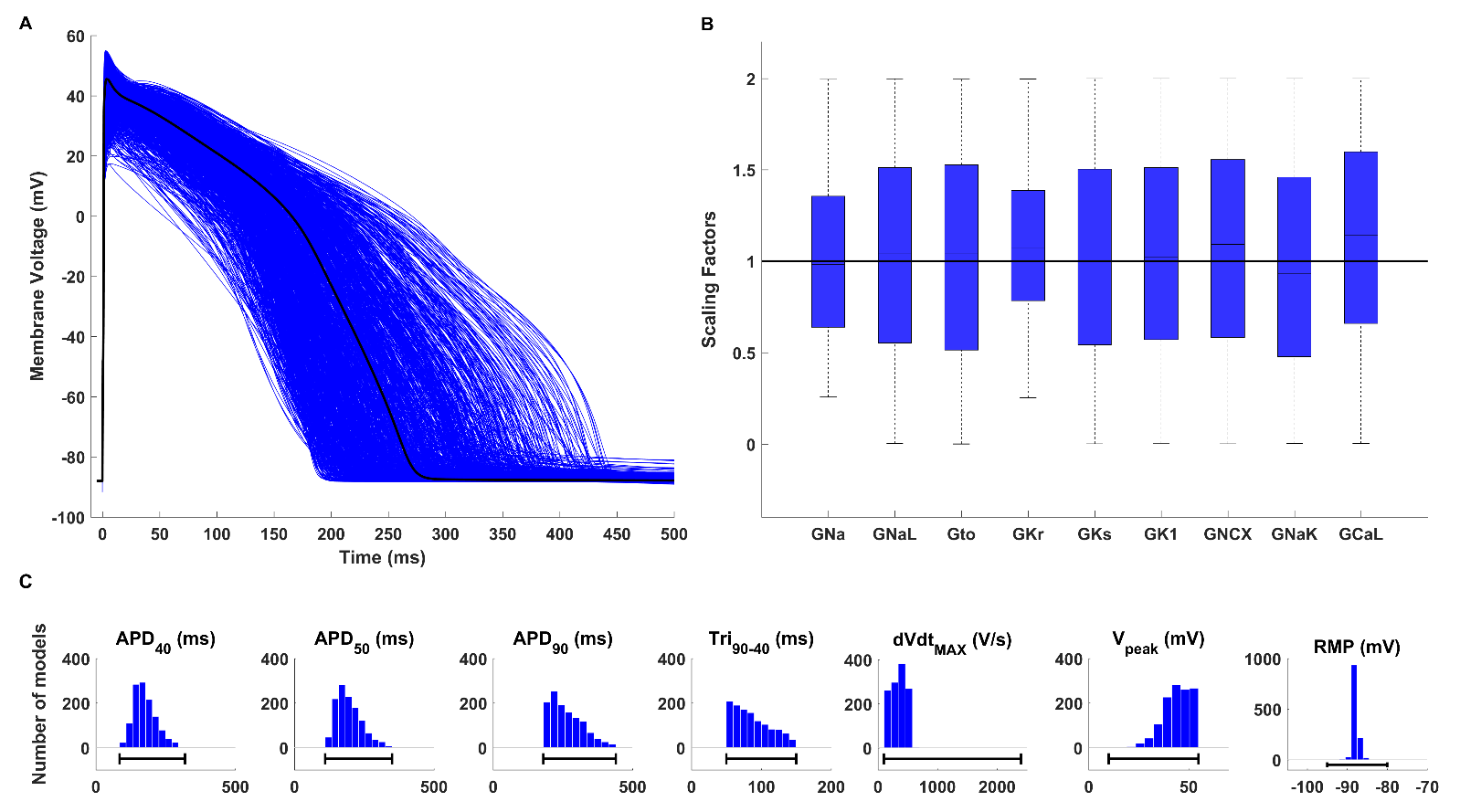


**Figure S1**. Characterisation of the population of human ventricular control models used for *in silico* drug trials. **(A)** AP traces of the 1,213 models accepted in the control population after the calibration with experimental human data (blue traces) and the baseline ORd model used to generate the population (black trace). **(B)** Distribution of ionic conductances in the calibrate population of human ventricular control models (blue boxplots). For each conductance, the values shown (between 0 and 2) represent the scaling factors of the 1,213 models in the population compared to the baseline model. All the scaling factors are equal to 1 for the ORd model (black line). On each box, the central mark is the median of the population, box limits are the 25^th^ and 75^th^ percentiles, and whiskers extend to the most extreme data points. **(C)** AP biomarker distributions for the calibrated population of models under control conditions (blue histograms) compared to the experimental ranges (black lines): AP duration at 40%, 50% and 90% of repolarisation (APD_40,_ APD_50,_ APD_90_); AP triangulation, defined as the difference between APD_90_ and APD_40_ (Tri_90-40_); maximum upstroke velocity (dV/dt_MAX_); peak voltage (V_peak_); resting membrane potential (RMP).

**
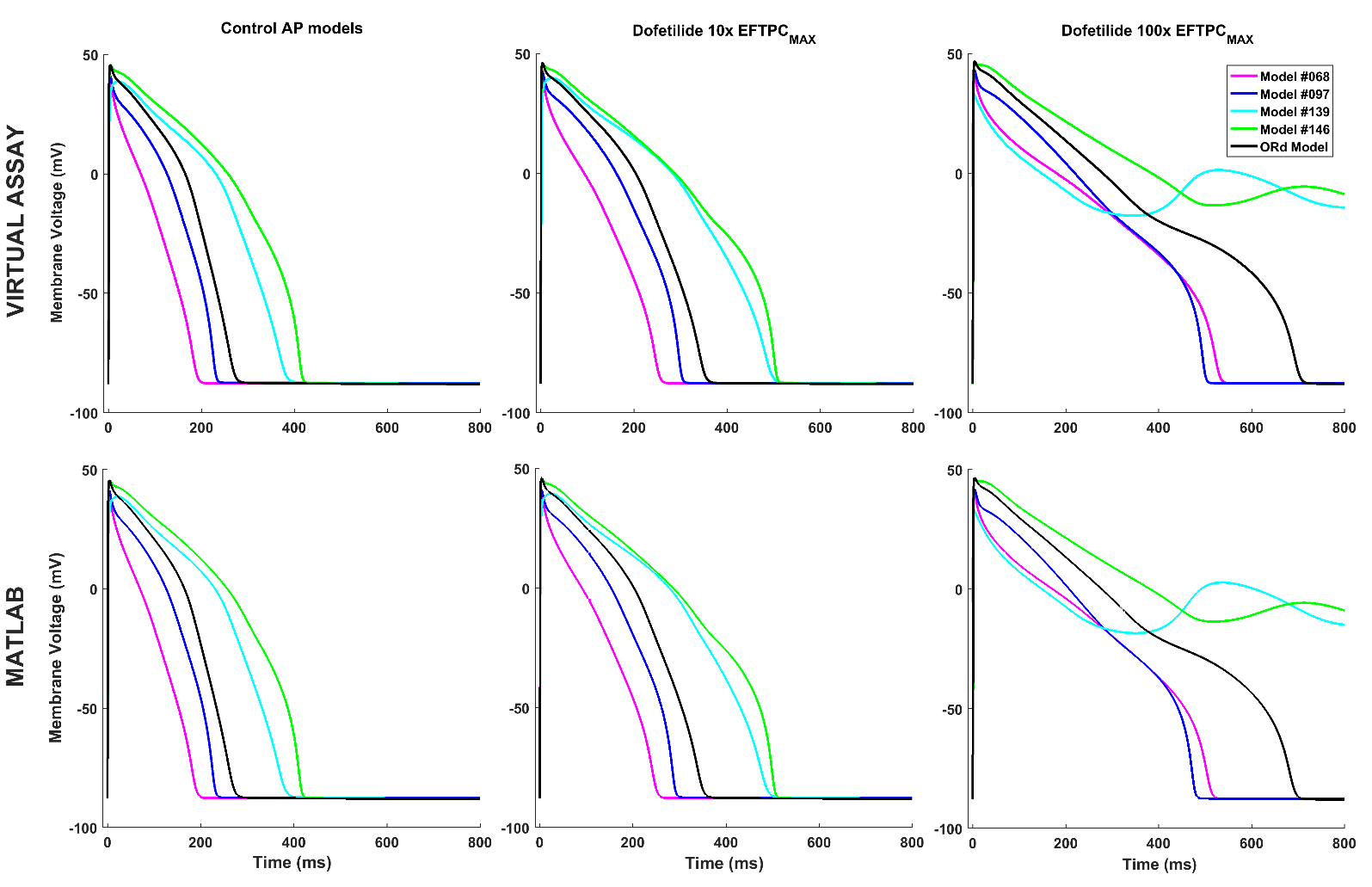
**

**Figure S2**. Comparison of the simulation results obtained with Virtual Assay (v.1.3.640 © 2014 Oxford University Innovation Ltd. Oxford, UK) and Matlab (Mathworks Inc. Natwick, MA, USA) for the ORd model and 4 additional models from the calibrated population of 1,213 human ventricular models. Simulations were compared both in control conditions and when considering the effect of Dofetilide I at two different concentrations (10x and 100x EFTPC_MAX_).


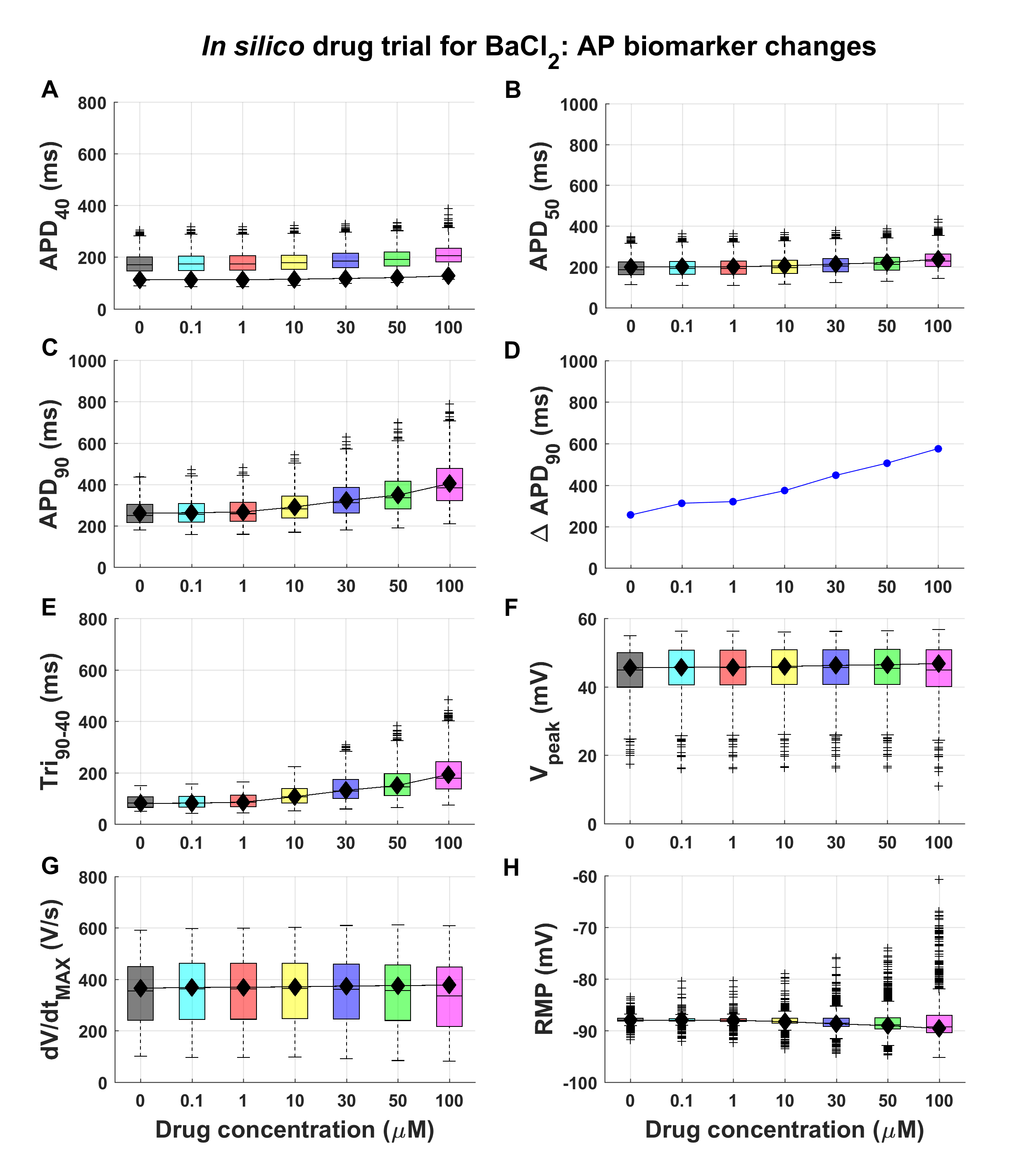


**Figure S3**. BaCl_2_ effect on 8 AP biomarkers: AP duration at 40%, 50% and 90% of repolarisation (APD_40,_ APD_50,_ APD_90_); APD_90_ dispersion (ΔAPD_90_, difference between the maximum and minimum value of APD_90_ in the population); AP triangulation (Tri_90-40_, difference between APD_90_ and APD_40_); maximum upstroke velocity (dV/dt_MAX_); peak voltage (V_peak_); resting membrane potential (RMP). Results are presented as boxplots showing the biomarker distributions in the population of human ventricular models. On each box, the central mark is the median of the population, box limits are the 25^th^ and 75^th^ percentiles, and whiskers extend to the most extreme data points not considered outliers, plotted individually as separate crosses. Results for the baseline ORd model are shown as filled black diamonds.


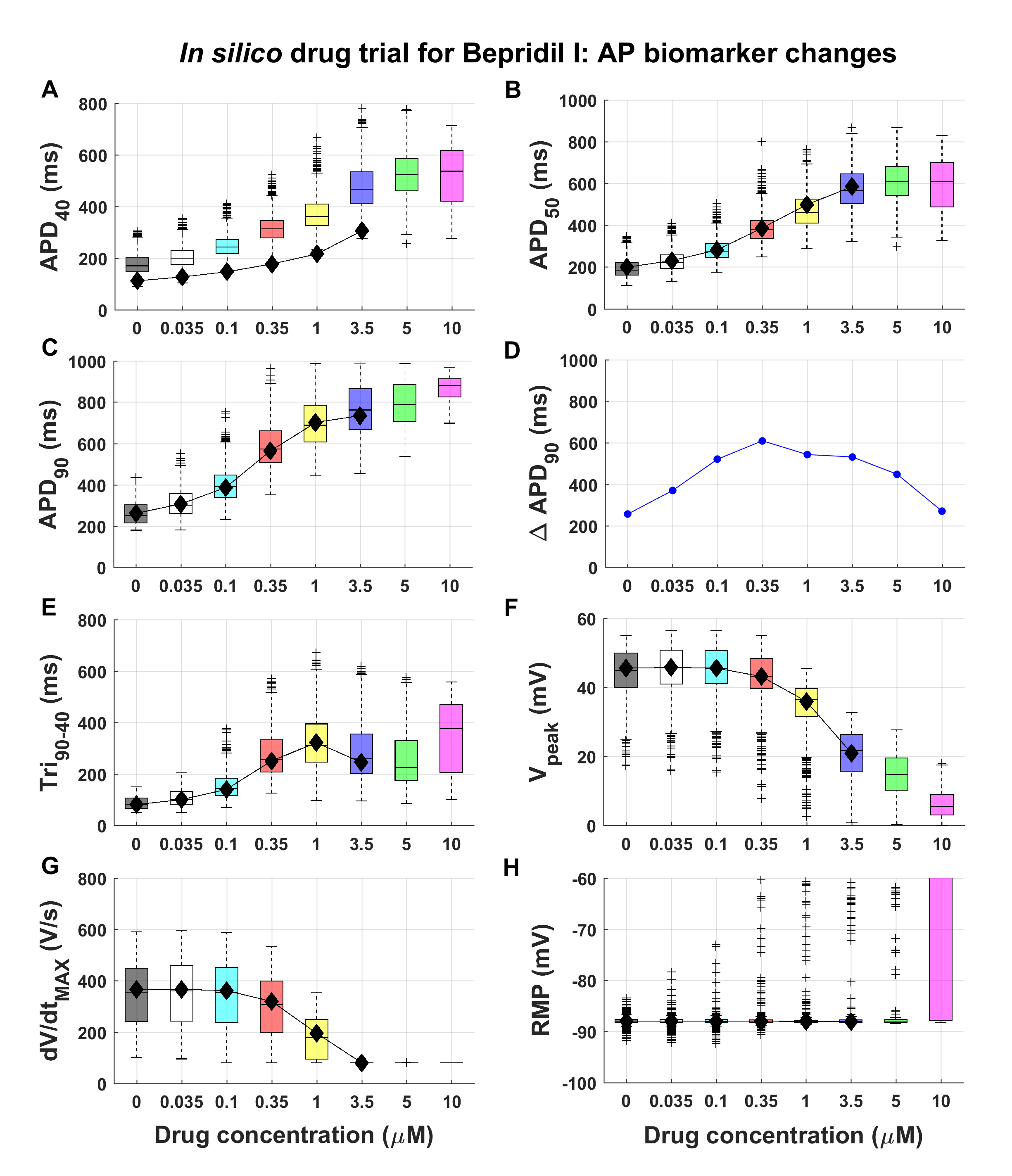


**Figure S4**. Bepridil I effect on 8 AP biomarkers. Results are presented as boxplots showing the AP biomarker distributions in the population of human ventricular models, while the results for the baseline ORd model are shown as filled black diamonds. Boxplot and AP biomarker descriptions as in Figure S3.


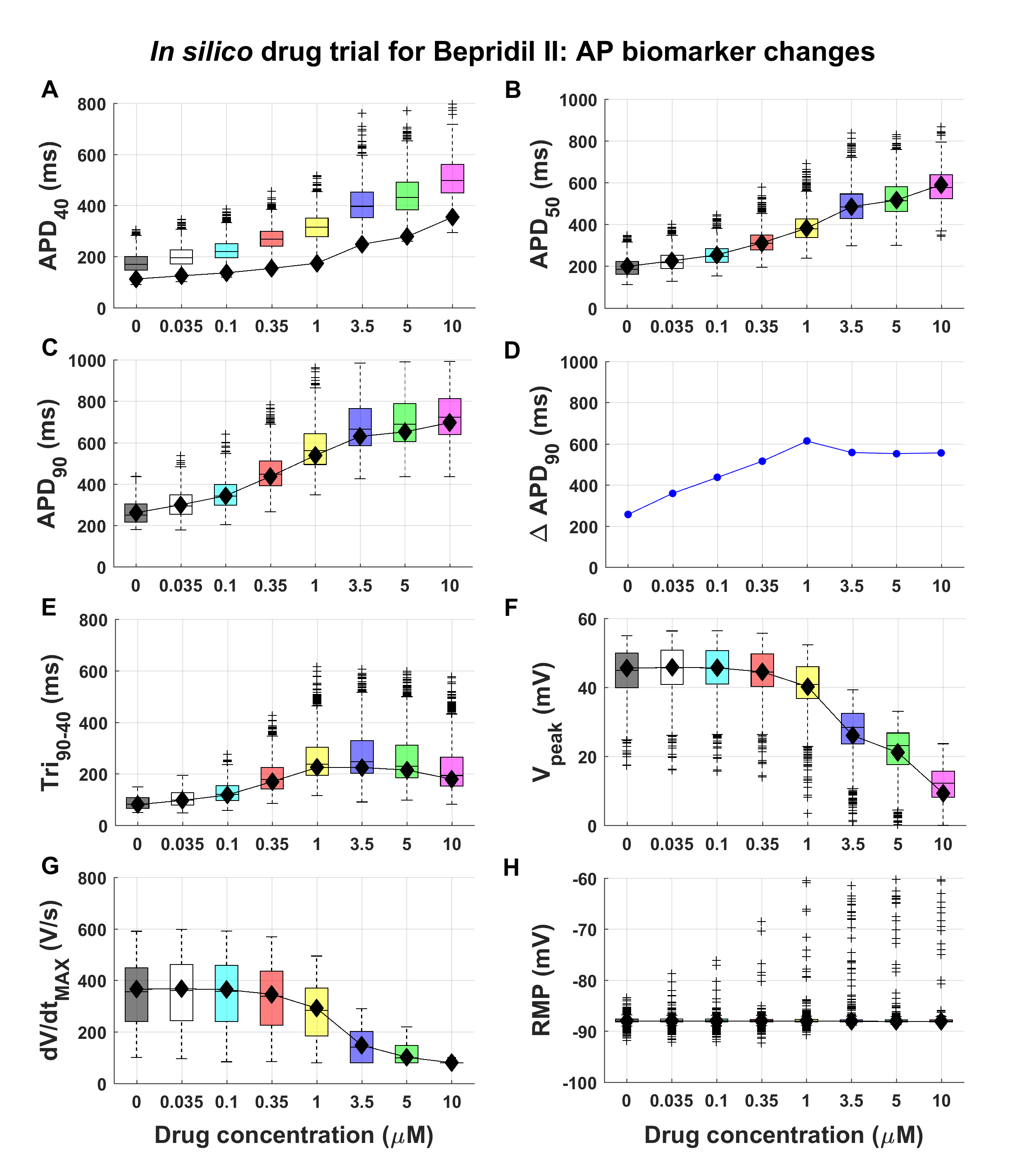


**Figure S5**. Bepridil II effect on 8 AP biomarkers. Results are presented as boxplots showing the AP biomarker distributions in the population of human ventricular models, while the results for the baseline ORd model are shown as filled black diamonds. Boxplot and AP biomarker descriptions as in Figure S3.


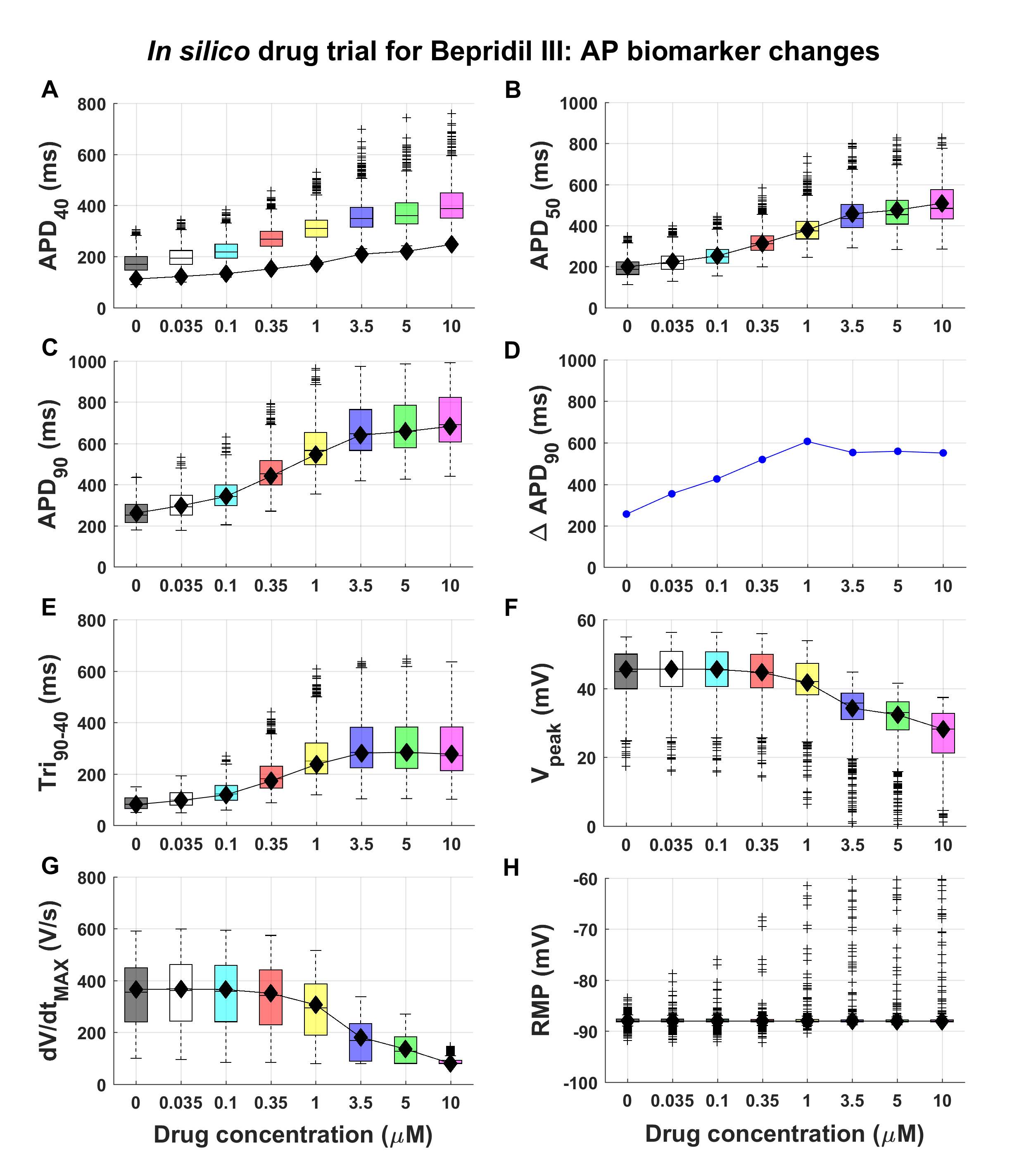


**Figure S6**. Bepridil III effect on 8 AP biomarkers. Results are presented as boxplots showing the AP biomarker distributions in the population of human ventricular models, while the results for the baseline ORd model are shown as filled black diamonds. Boxplot and AP biomarker descriptions as in Figure S3.


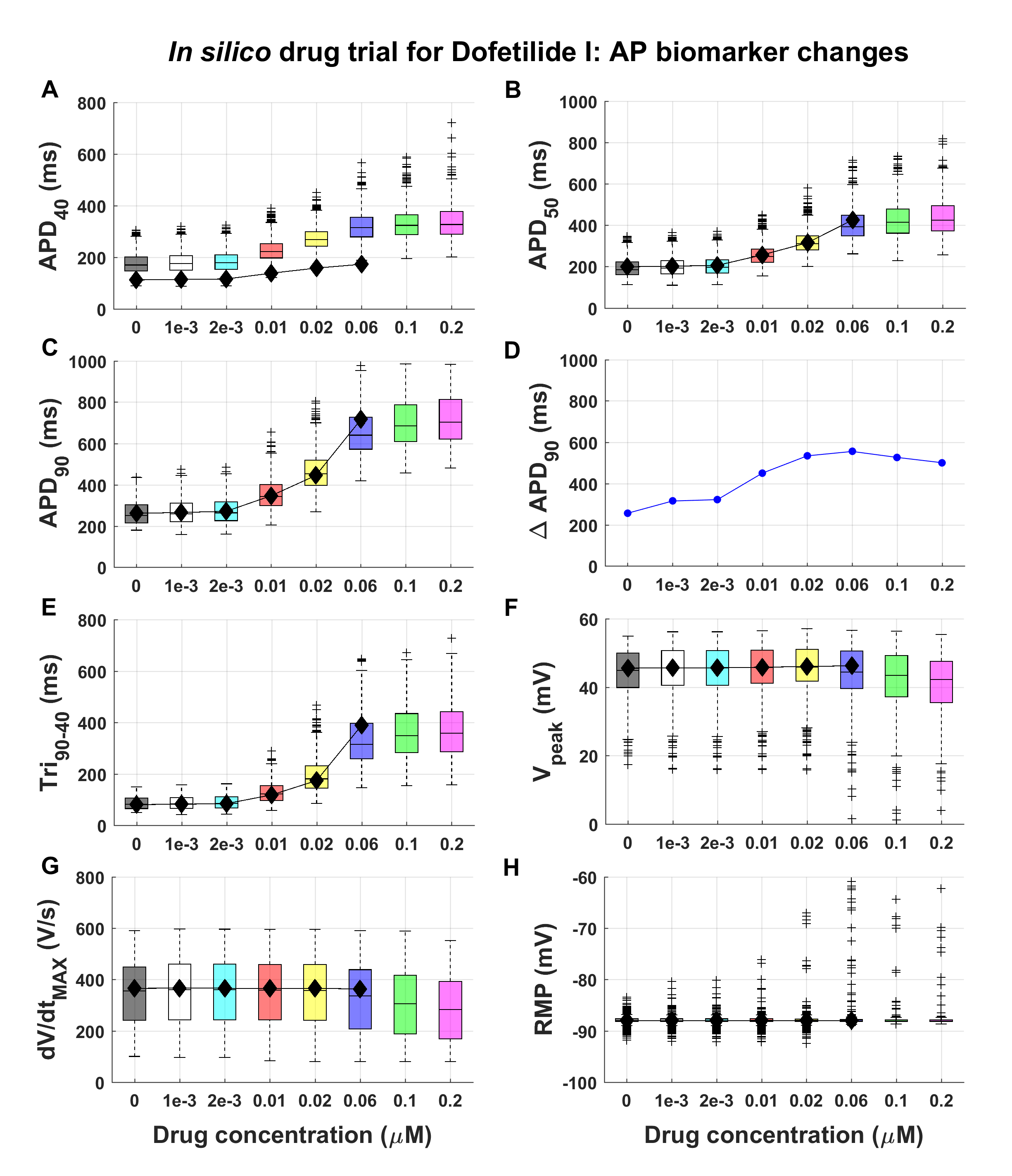


**Figure S7**. Dofetilide I effect on 8 AP biomarkers. Results are presented as boxplots showing the AP biomarker distributions in the population of human ventricular models, while the results for the baseline ORd model are shown as filled black diamonds. Boxplot and AP biomarker descriptions as in Figure S3.


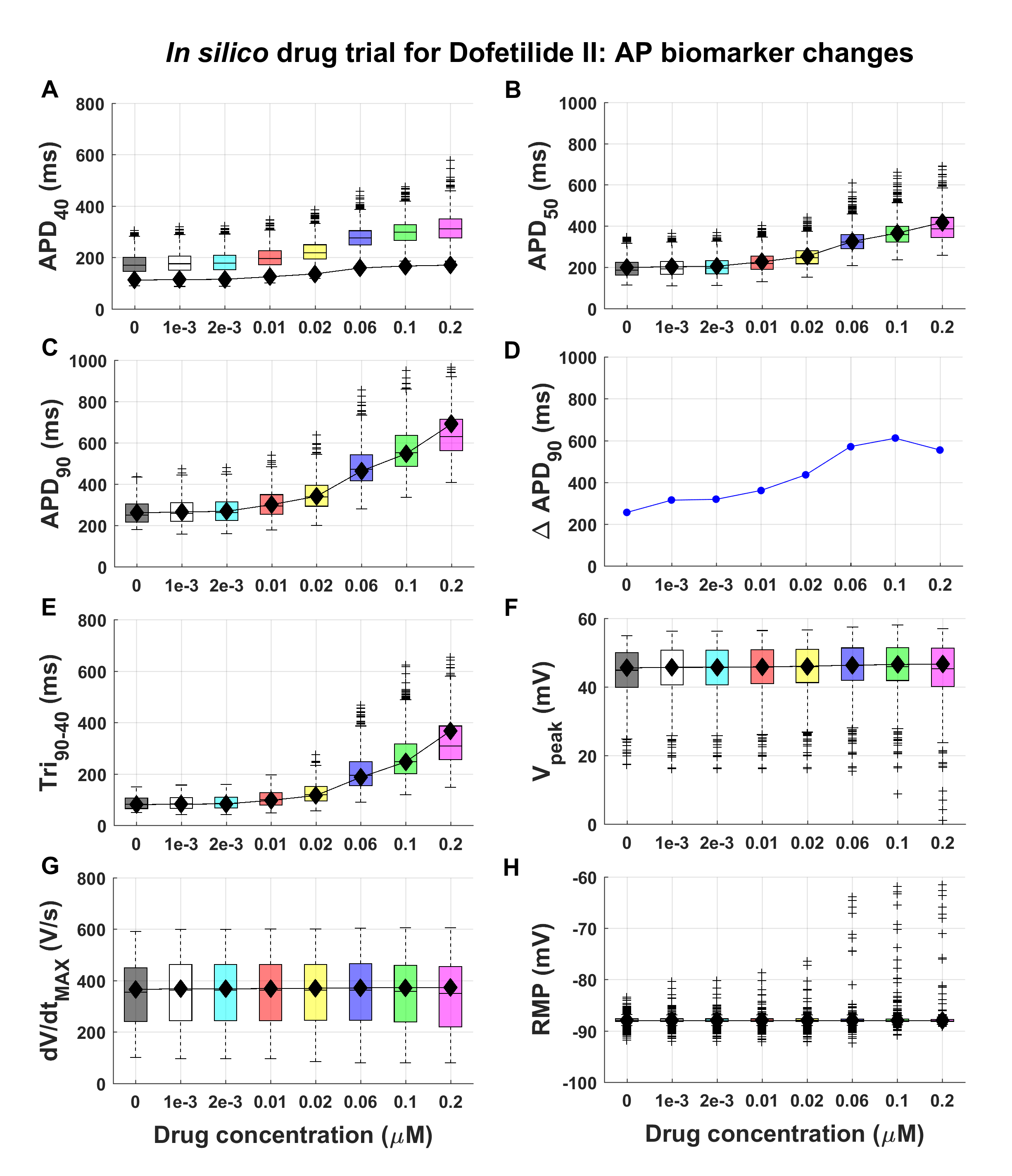


**Figure S8**. Dofetilide II effect on 8 AP biomarkers. Results are presented as boxplots showing the AP biomarker distributions in the population of human ventricular models, while the results for the baseline ORd model are shown as filled black diamonds. Boxplot and AP biomarker descriptions as in Figure S3.


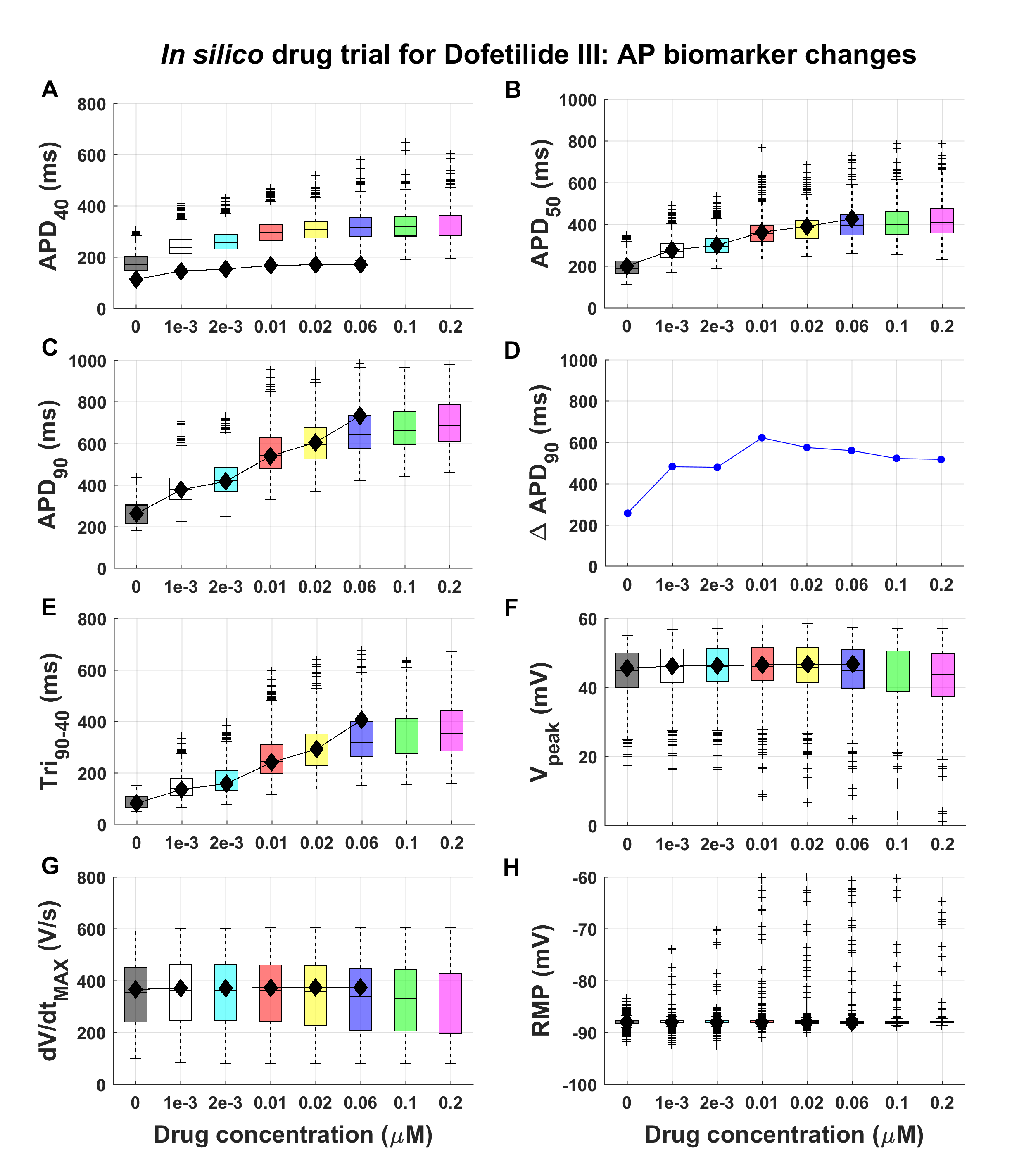


**Figure S9**. Dofetilide III effect on 8 AP biomarkers. Results are presented as boxplots showing the AP biomarker distributions in the population of human ventricular models, while the results for the baseline ORd model are shown as filled black diamonds. Boxplot and AP biomarker descriptions as in Figure S3.

**Figure S10**. Flecainide I effect on 8 AP biomarkers. Results are presented as boxplots showing the AP biomarker distributions in the population of human ventricular models, while the results for the baseline ORd model are shown as filled black diamonds. Boxplot and AP biomarker descriptions as in Figure S3.


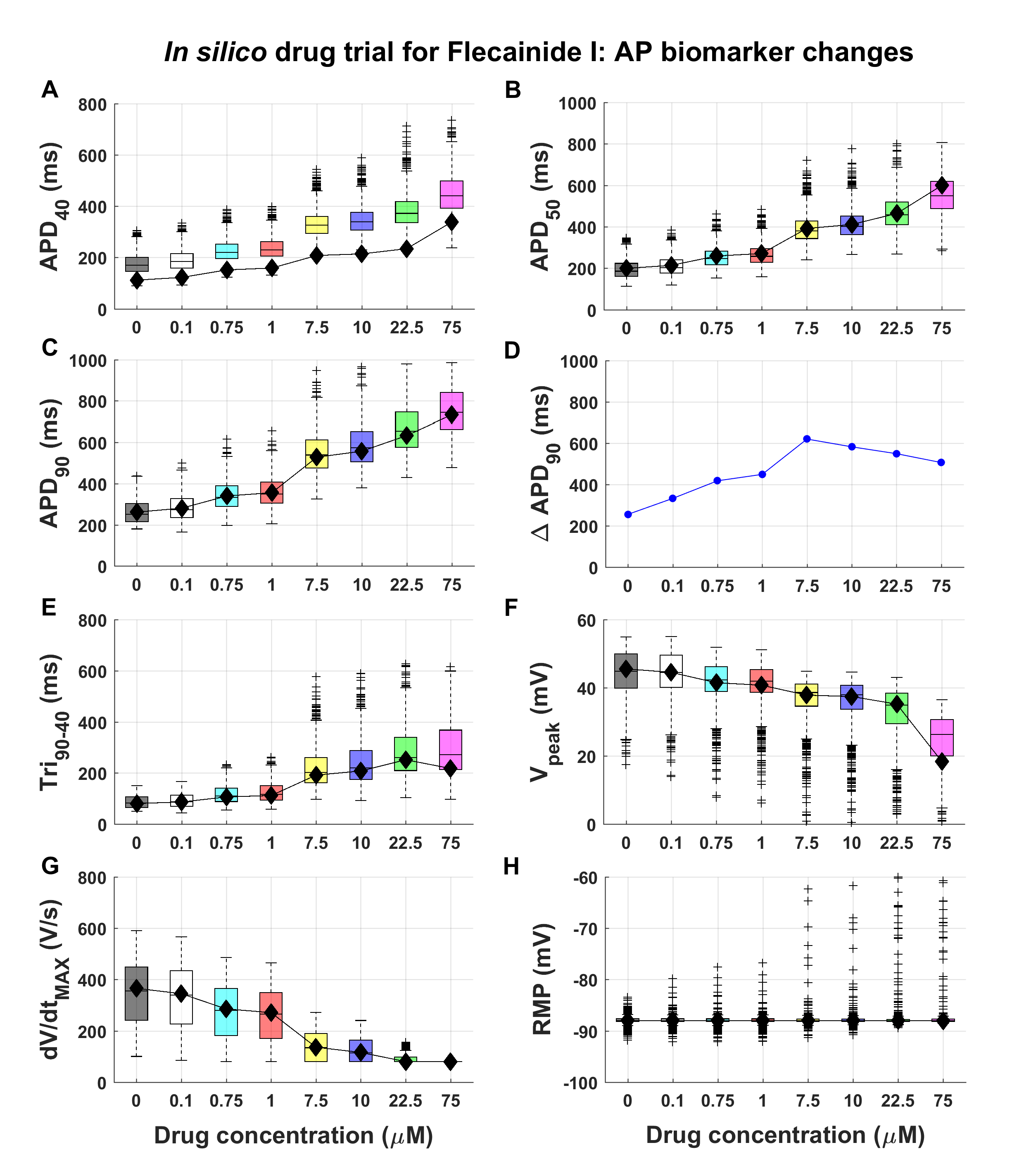

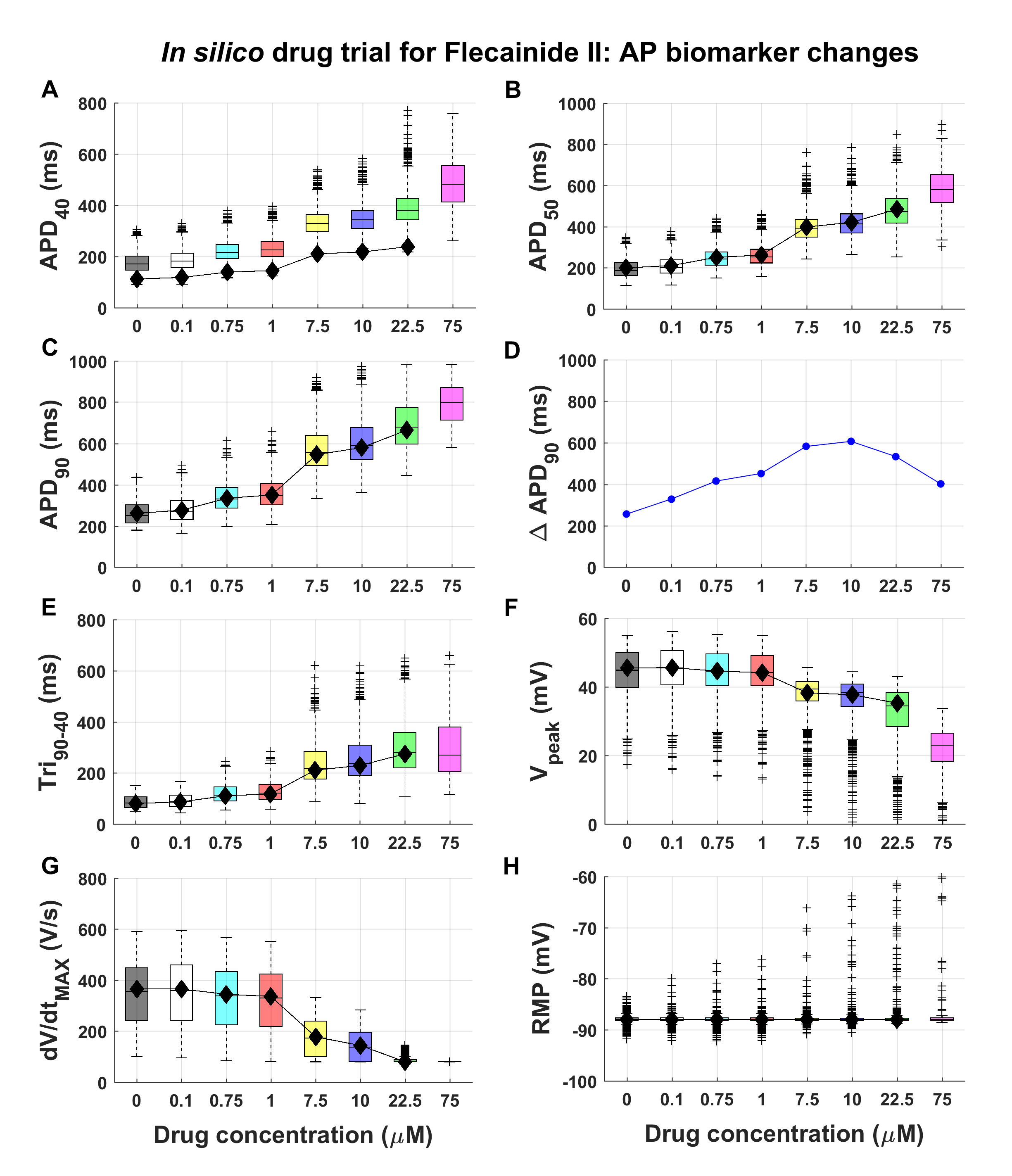


**Figure S11**. Flecainide II effect on 8 AP biomarkers. Results are presented as boxplots showing the AP biomarker distributions in the population of human ventricular models, while the results for the baseline ORd model are shown as filled black diamonds. Boxplot and AP biomarker descriptions as in Figure S3.


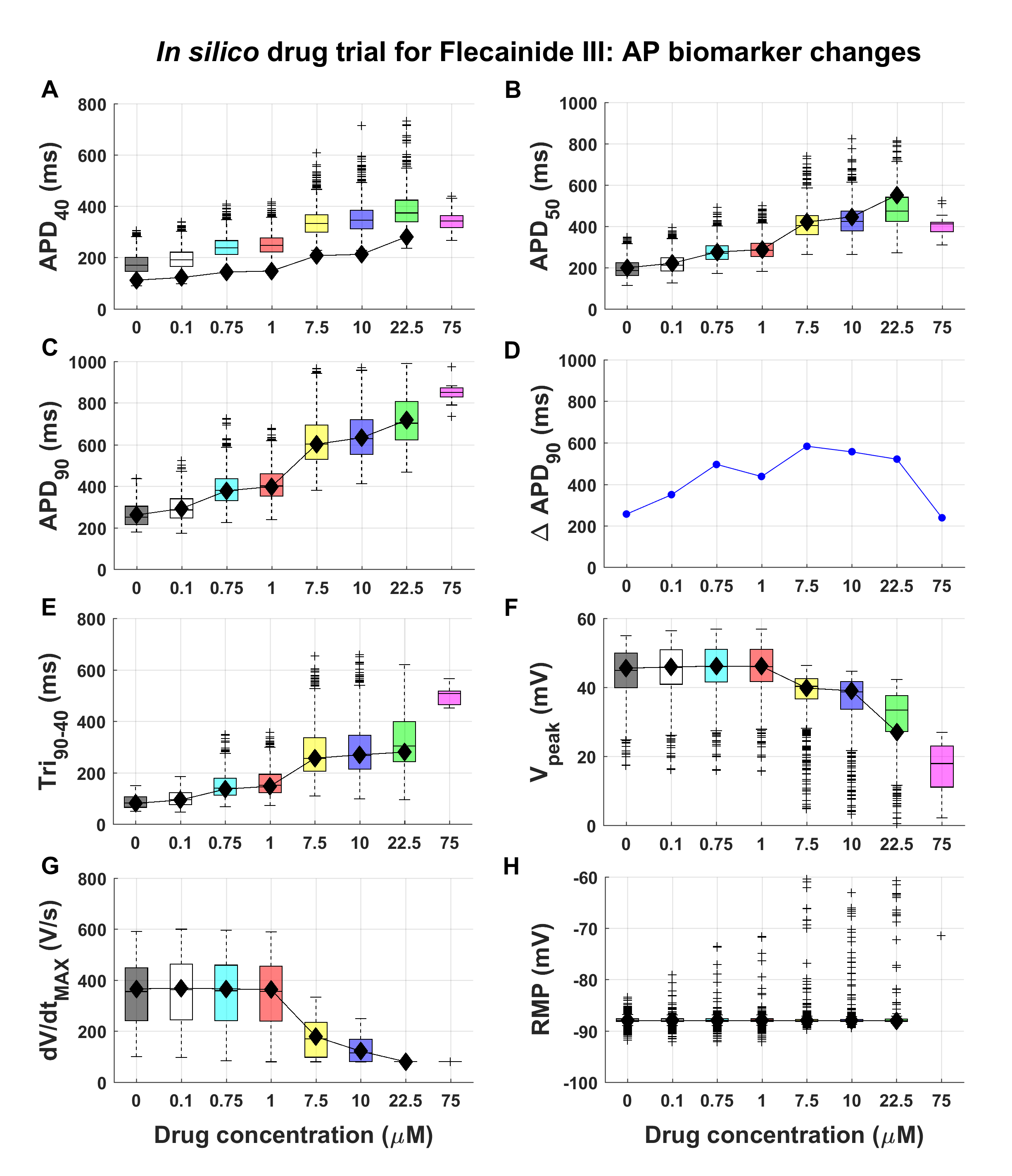


**Figure S12**. Flecainide III effect on 8 AP biomarkers. Results are presented as boxplots showing the AP biomarker distributions in the population of human ventricular models, while the results for the baseline ORd model are shown as filled black diamonds. Boxplot and AP biomarker descriptions as in Figure S3.


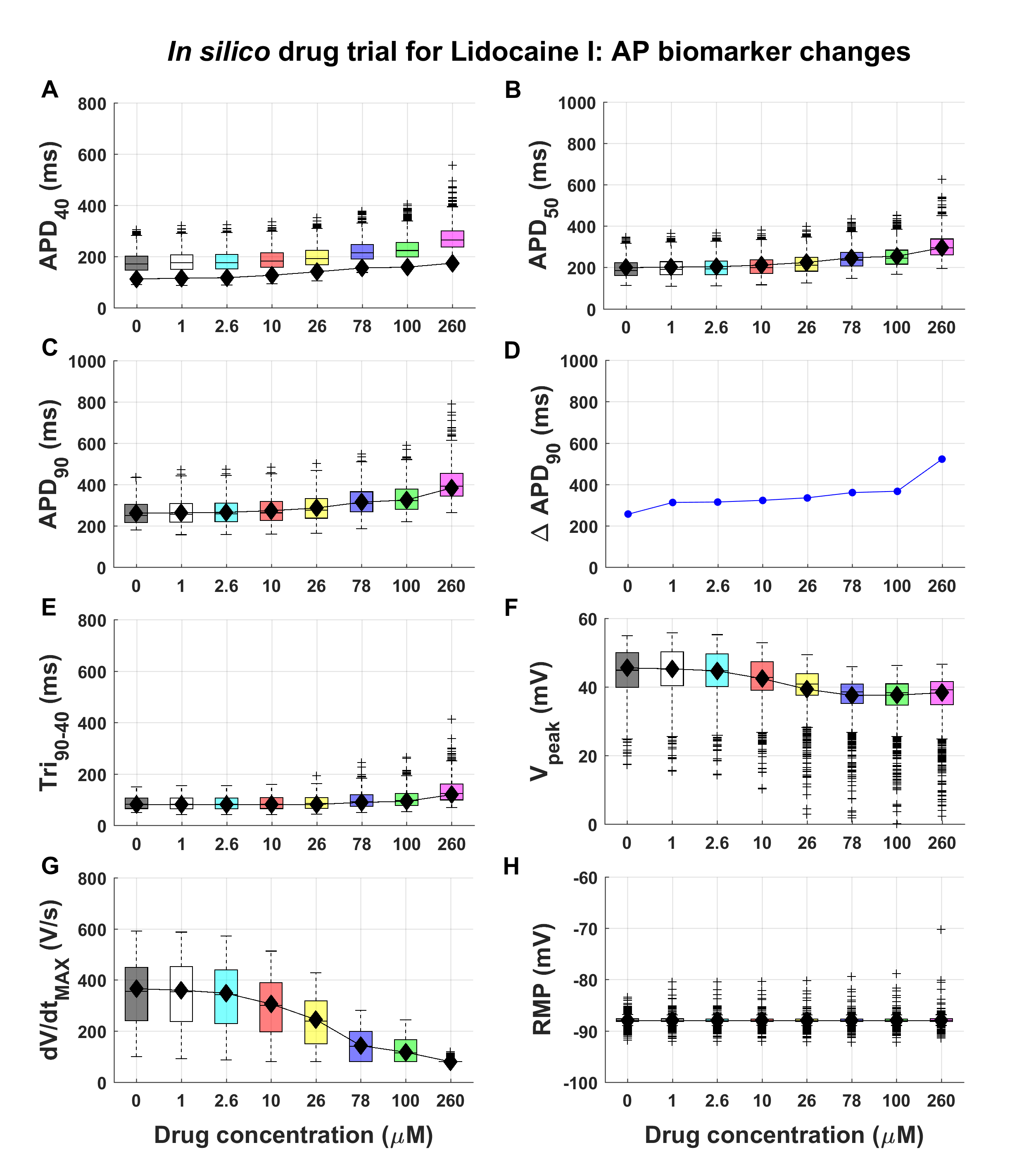


**Figure S13**. Lidocaine I effect on 8 AP biomarkers. Results are presented as boxplots showing the AP biomarker distributions in the population of human ventricular models, while the results for the baseline ORd model are shown as filled black diamonds. Boxplot and AP biomarker descriptions as in Figure S3.


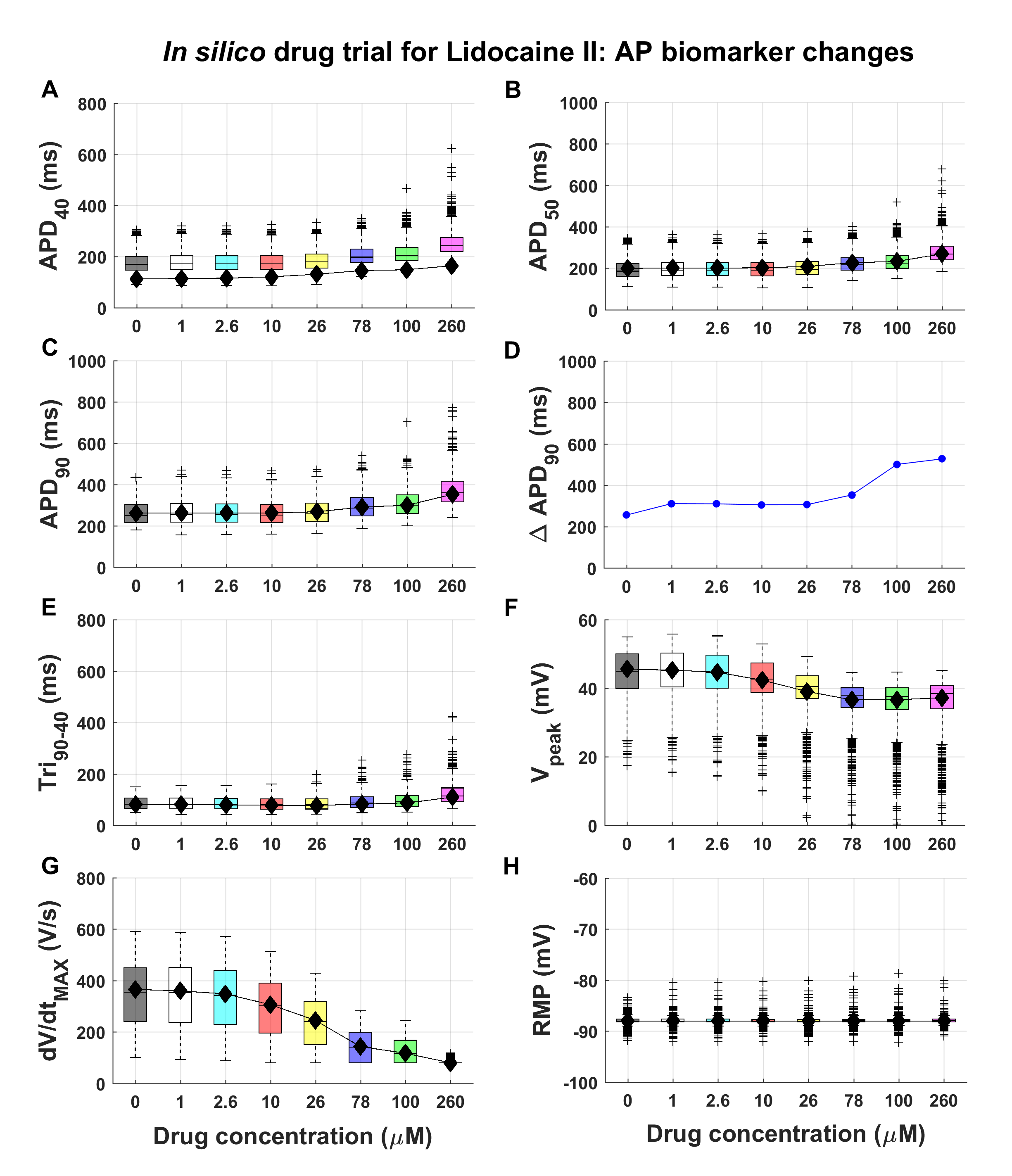


**Figure S14**. Lidocaine II effect on 8 AP biomarkers. Results are presented as boxplots showing the AP biomarker distributions in the population of human ventricular models, while the results for the baseline ORd model are shown as filled black diamonds. Boxplot and AP biomarker descriptions as in Figure S3.

**Figure S15**. Mexiletine I effect on 8 AP biomarkers. Results are presented as boxplots showing the AP biomarker distributions in the population of human ventricular models, while the results for the baseline ORd model are shown as filled black diamonds. Boxplot and AP biomarker descriptions as in Figure S3.


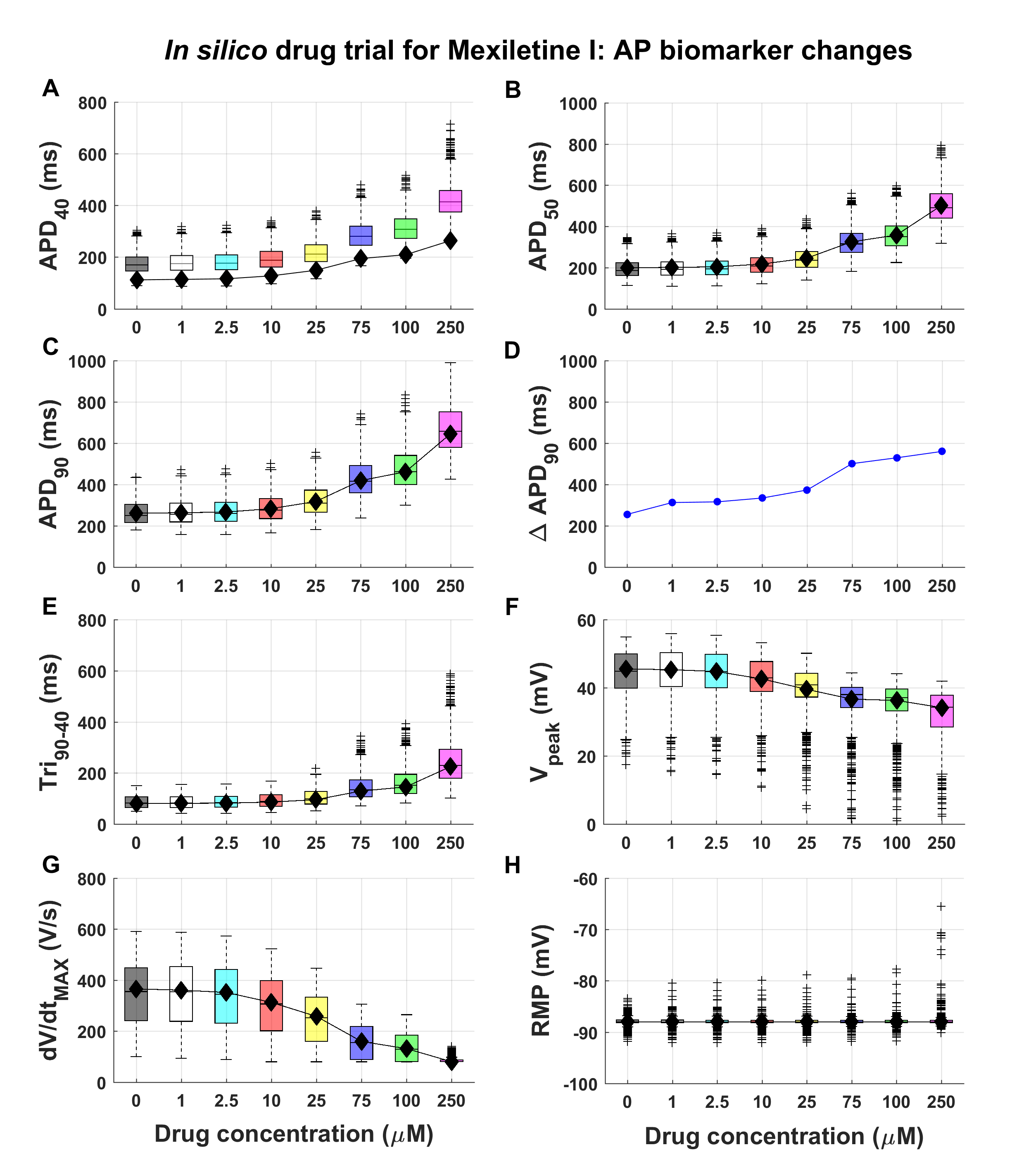

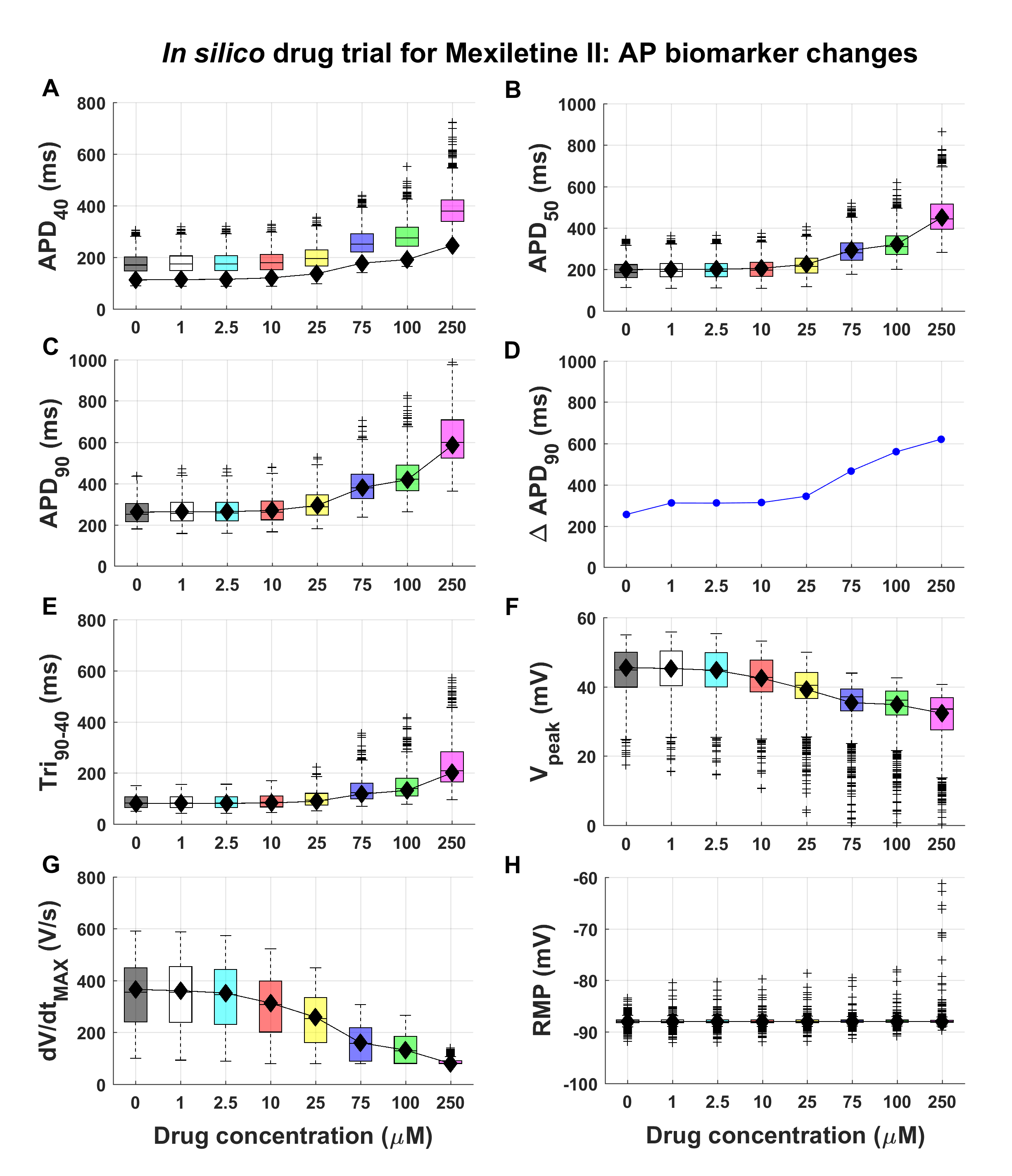


**Figure S16**. Mexiletine II effect on 8 AP biomarkers. Results are presented as boxplots showing the AP biomarker distributions in the population of human ventricular models, while the results for the baseline ORd model are shown as filled black diamonds. Boxplot and AP biomarker descriptions as in Figure S3.


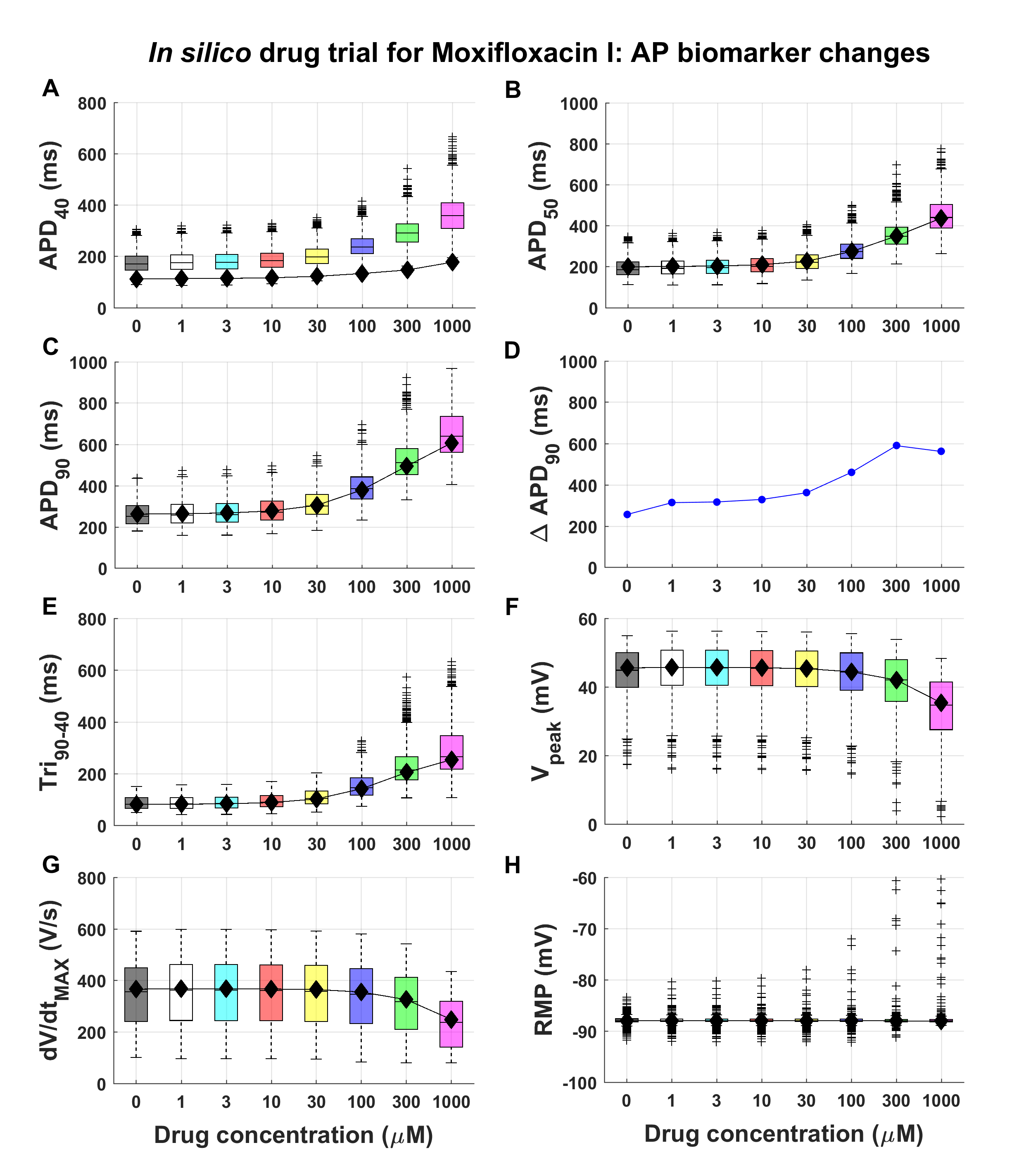


**Figure S17**. Moxifloxacin I effect on 8 AP biomarkers. Results are presented as boxplots showing the AP biomarker distributions in the population of human ventricular models, while the results for the baseline ORd model are shown as filled black diamonds. Boxplot and AP biomarker descriptions as in Figure S3.


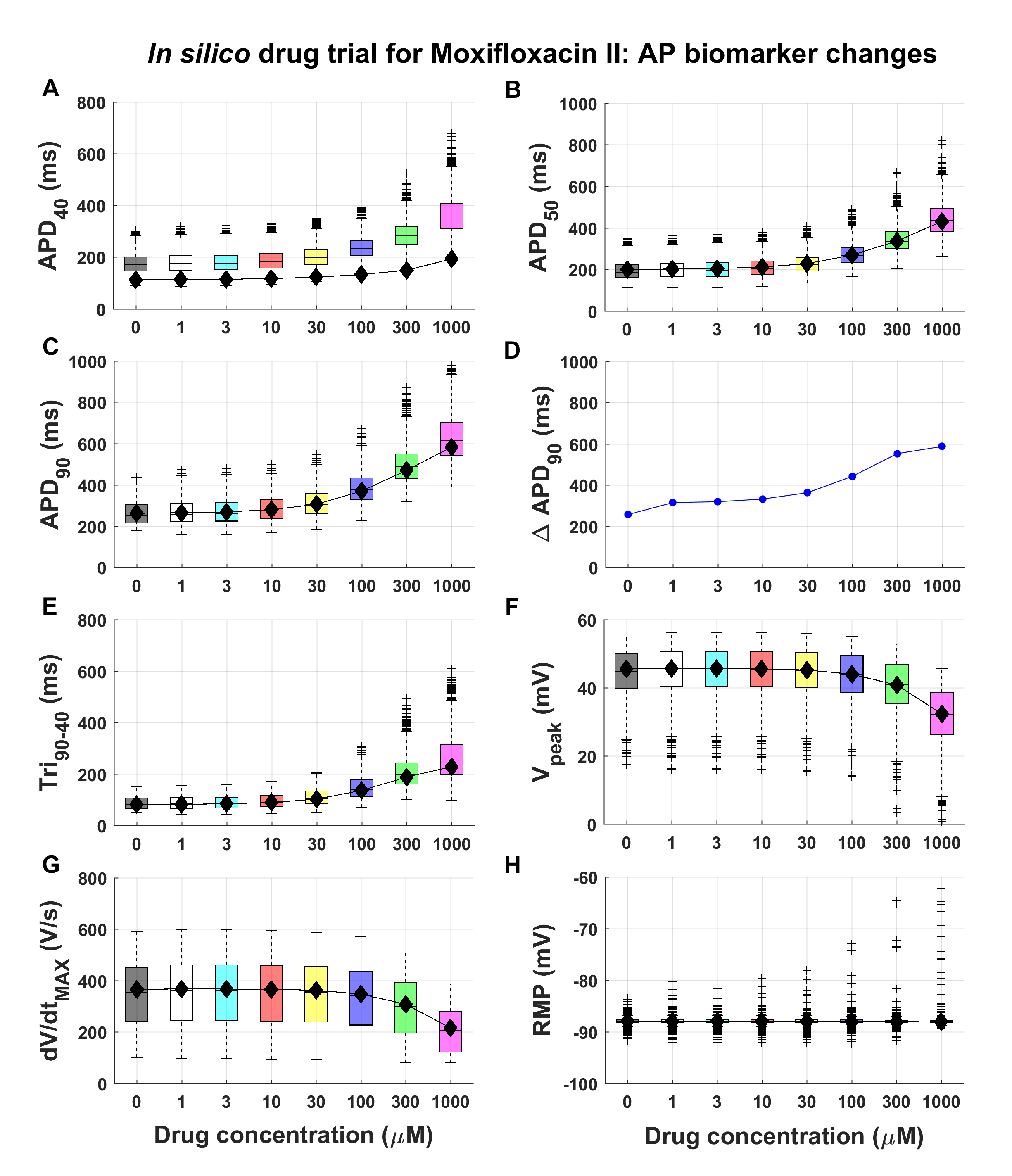


**Figure S18**. Moxifloxacin II effect on 8 AP biomarkers. Results are presented as boxplots showing the AP biomarker distributions in the population of human ventricular models, while the results for the baseline ORd model are shown as filled black diamonds. Boxplot and AP biomarker descriptions as in Figure S3.

**Figure S19**. Moxifloxacin III effect on 8 AP biomarkers. Results are presented as boxplots showing the AP biomarker distributions in the population of human ventricular models, while the results for the baseline ORd model are shown as filled black diamonds. Boxplot and AP biomarker descriptions as in Figure S3.


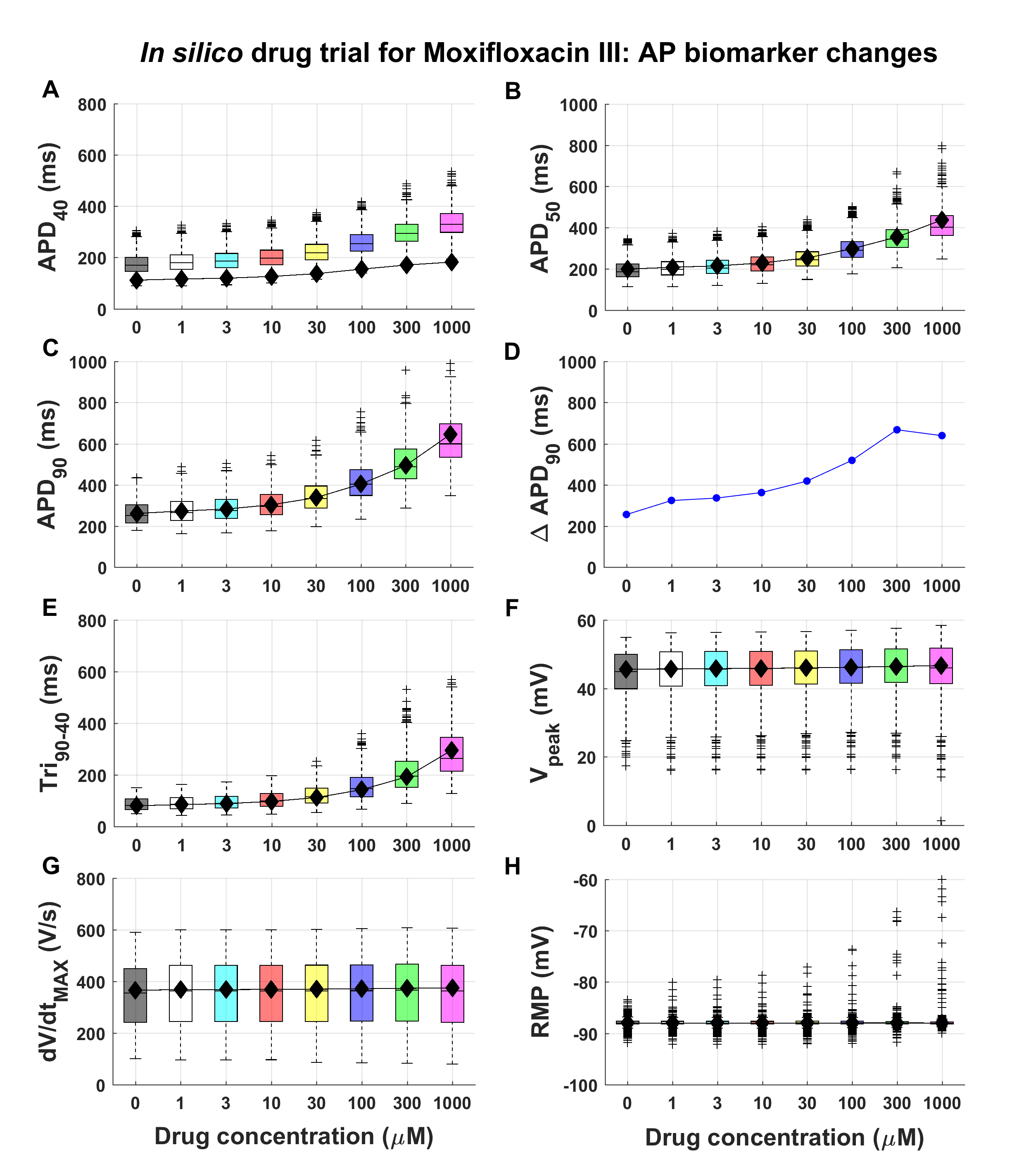

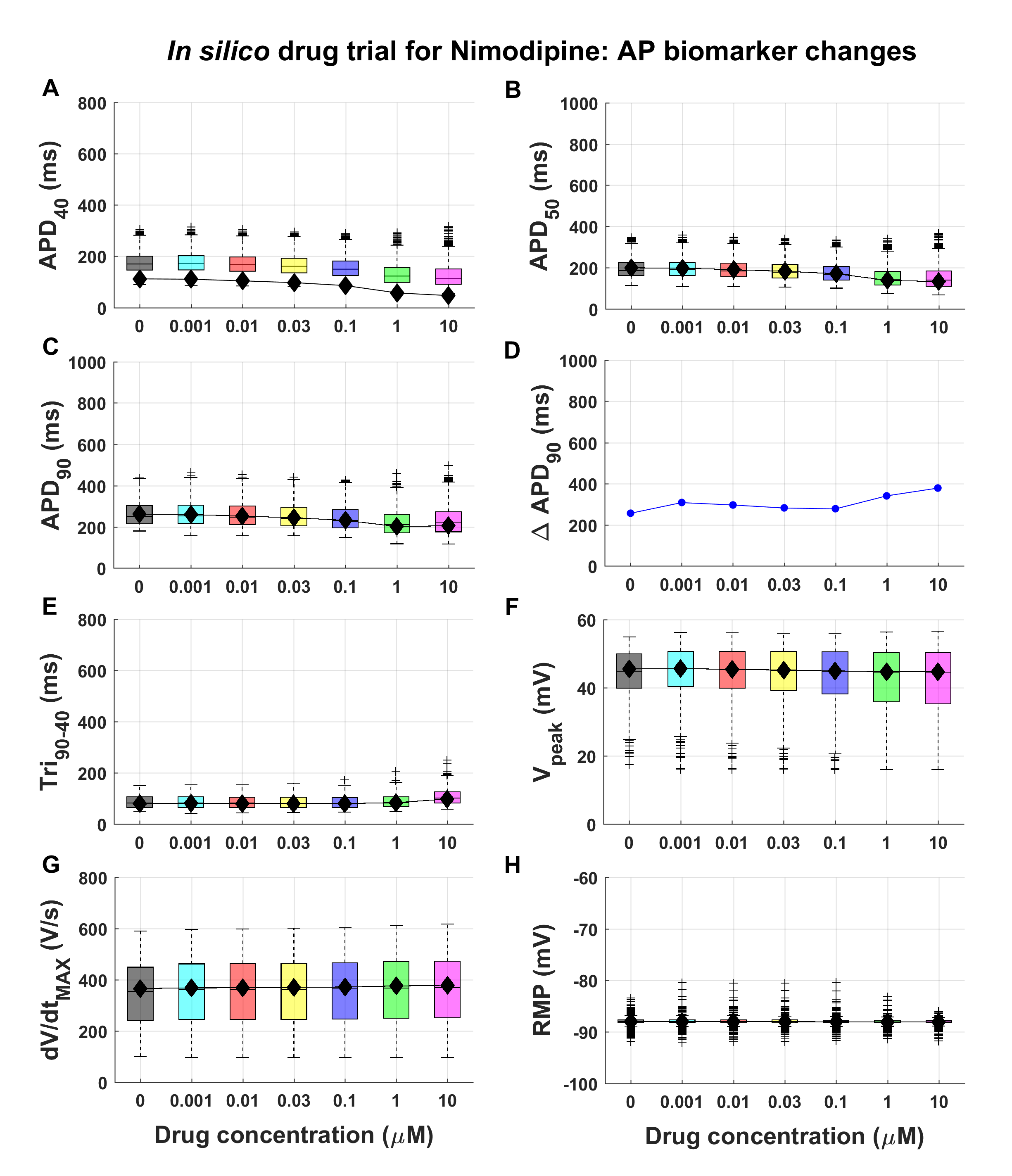


**Figure S20**. Nimodipine effect on 8 AP biomarkers. Results are presented as boxplots showing the AP biomarker distributions in the population of human ventricular models, while the results for the baseline ORd model are shown as filled black diamonds. Boxplot and AP biomarker descriptions as in Figure S3.


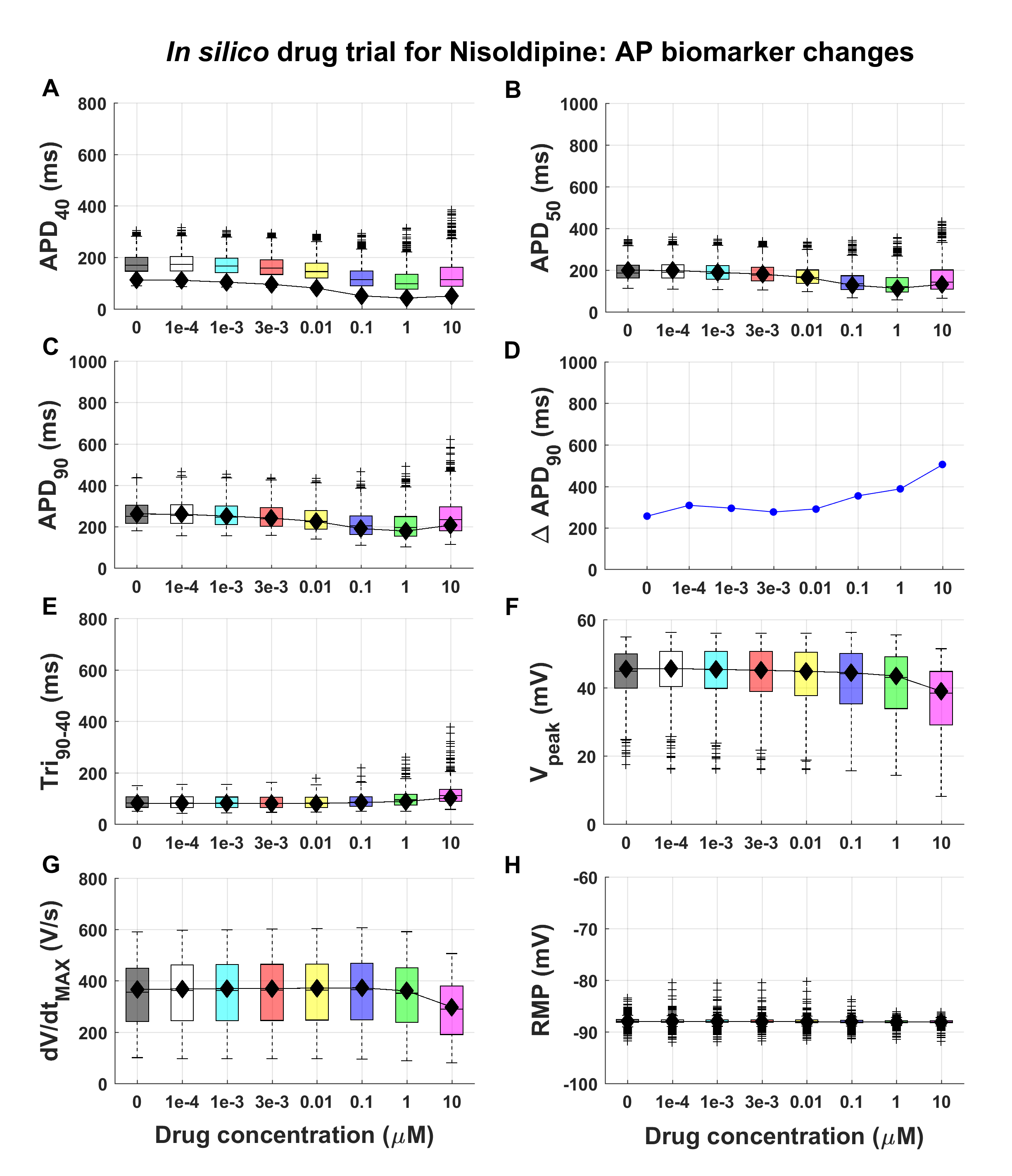


**Figure S21**. Nisoldipine effect on 8 AP biomarkers. Results are presented as boxplots showing the AP biomarker distributions in the population of human ventricular models, while the results for the baseline ORd model are shown as filled black diamonds. Boxplot and AP biomarker descriptions as in Figure S3.


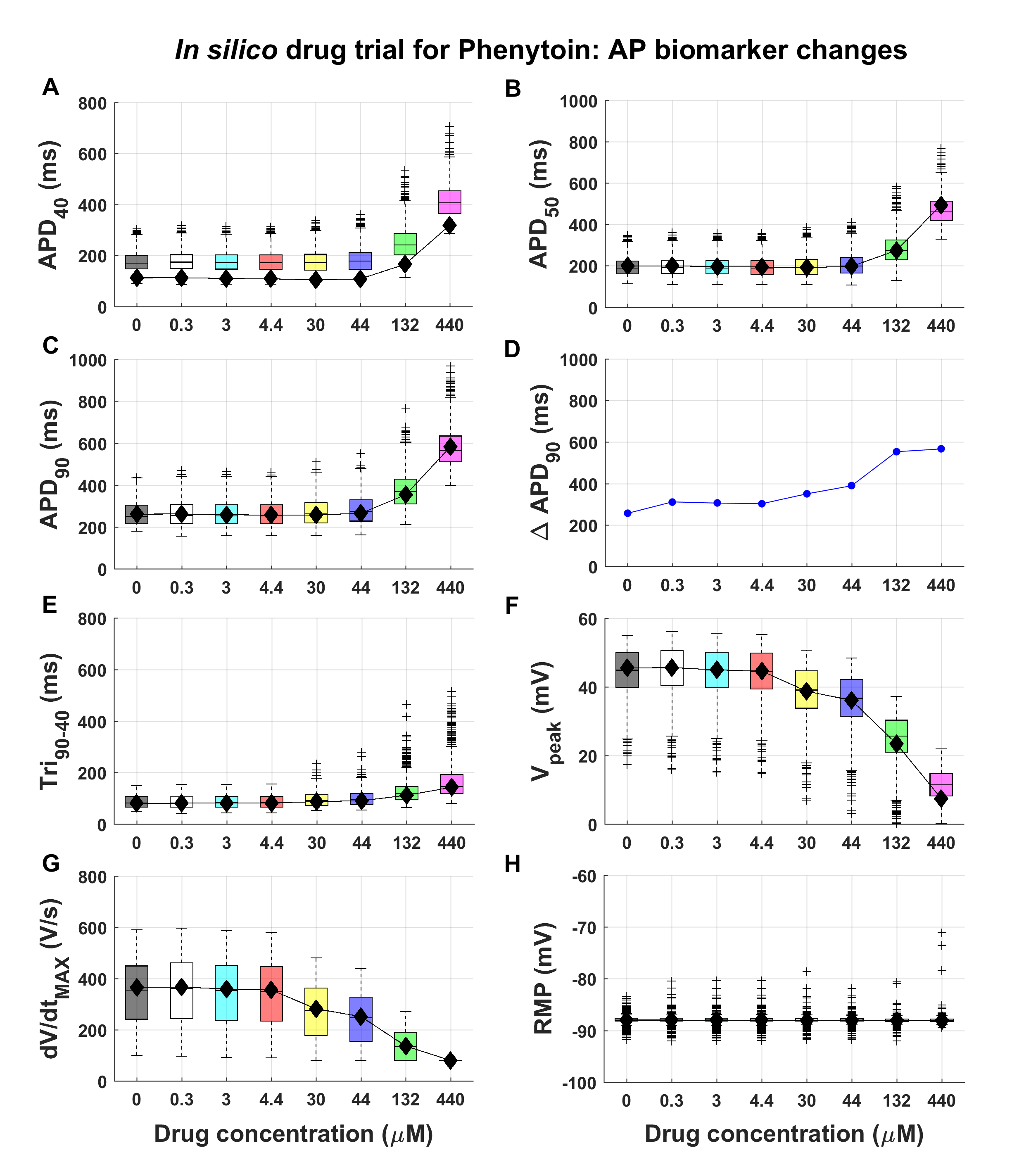


**Figure S22**. Phenytoin effect on 8 AP biomarkers. Results are presented as boxplots showing the AP biomarker distributions in the population of human ventricular models, while the results for the baseline ORd model are shown as filled black diamonds. Boxplot and AP biomarker descriptions as in Figure S3.


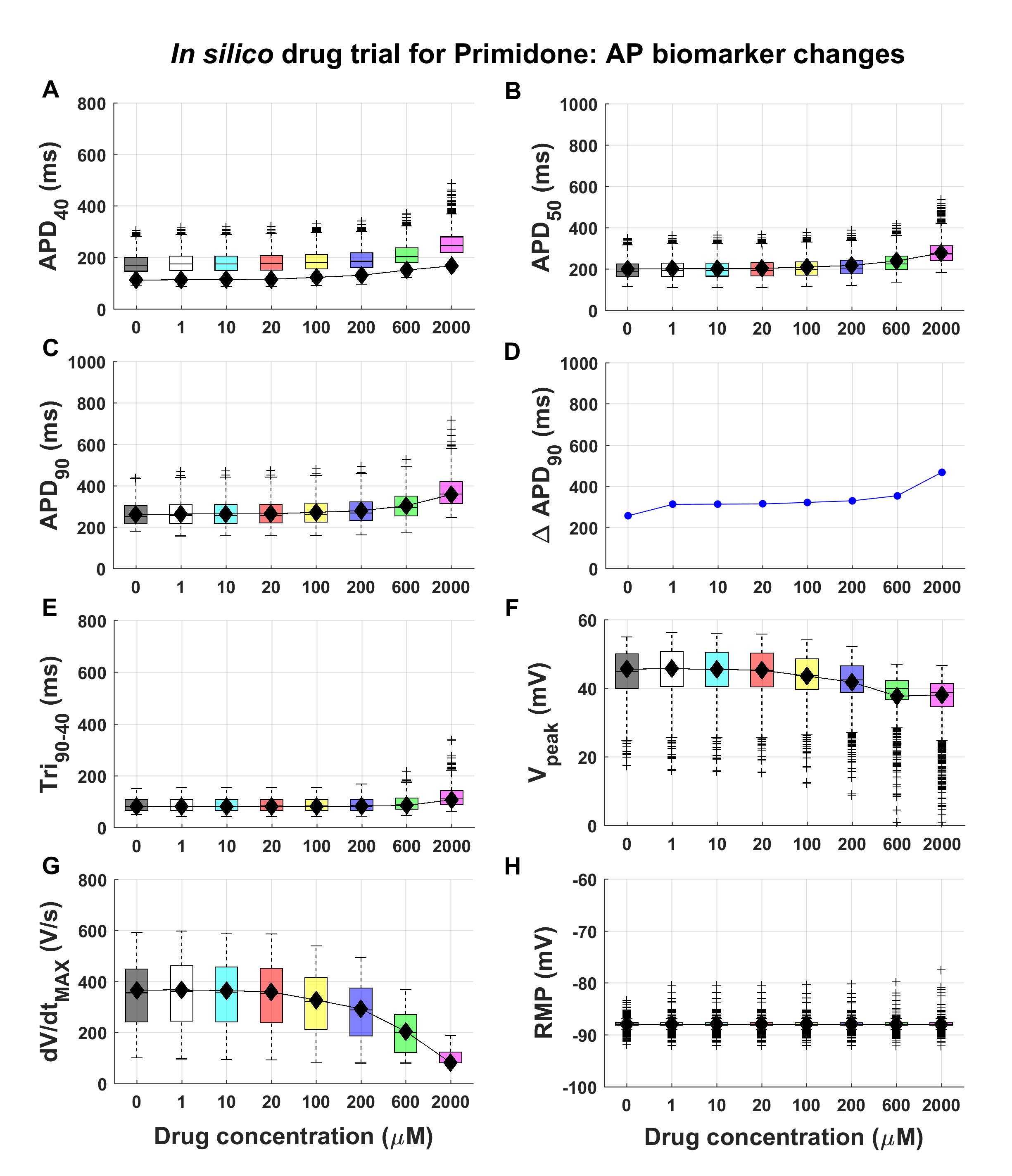


**Figure S23**. Primidone effect on 8 AP biomarkers. Results are presented as boxplots showing the AP biomarker distributions in the population of human ventricular models, while the results for the baseline ORd model are shown as filled black diamonds. Boxplot and AP biomarker descriptions as in Figure S3.


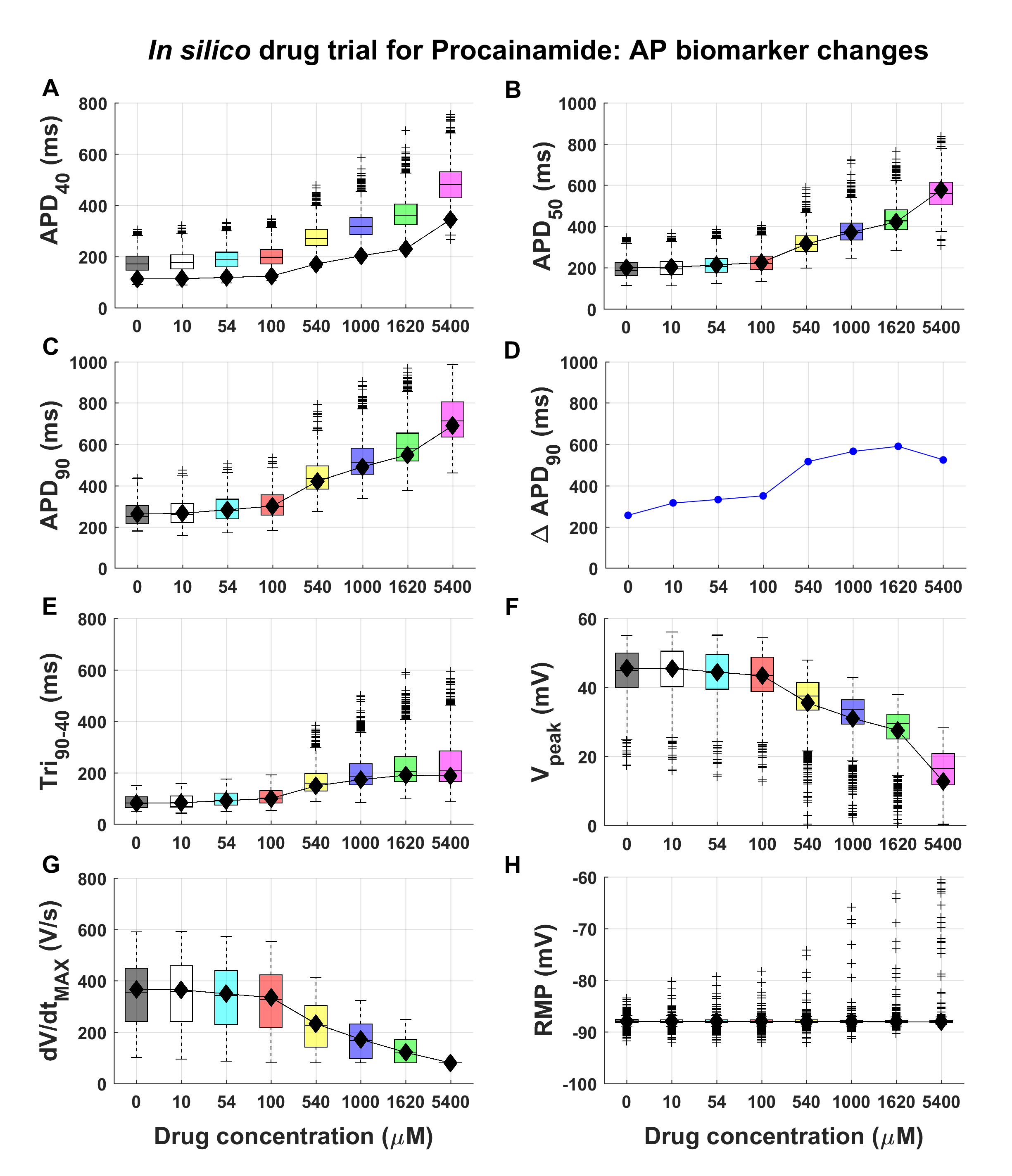


**Figure S24**. Procainamide effect on 8 AP biomarkers. Results are presented as boxplots showing the AP biomarker distributions in the population of human ventricular models, while the results for the baseline ORd model are shown as filled black diamonds. Boxplot and AP biomarker descriptions as in Figure S3.


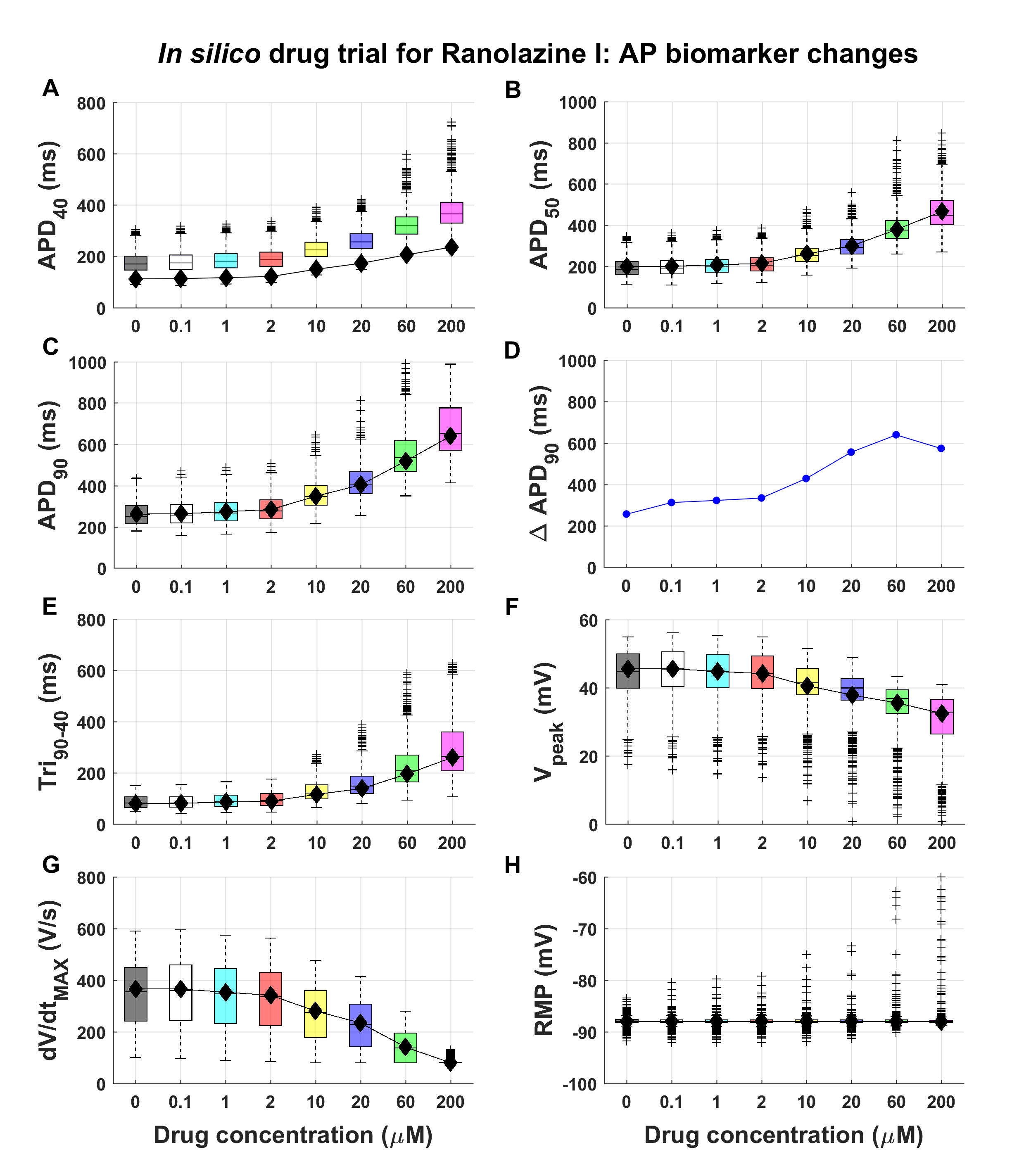


**Figure S25**. Ranolazine I effect on 8 AP biomarkers. Results are presented as boxplots showing the AP biomarker distributions in the population of human ventricular models, while the results for the baseline ORd model are shown as filled black diamonds. Boxplot and AP biomarker descriptions as in Figure S3.


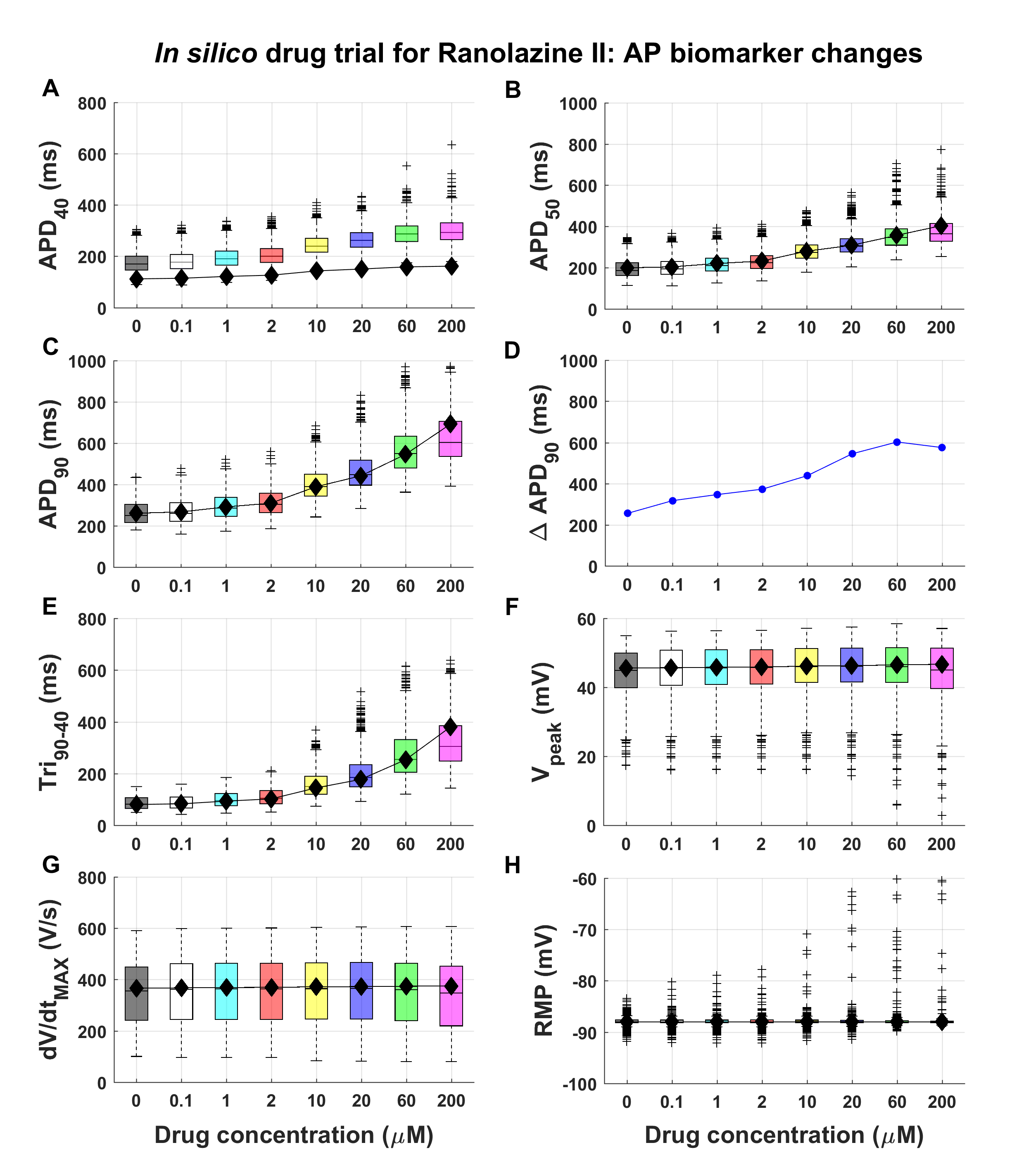


**Figure S26**. Ranolazine II effect on 8 AP biomarkers. Results are presented as boxplots showing the AP biomarker distributions in the population of human ventricular models, while the results for the baseline ORd model are shown as filled black diamonds. Boxplot and AP biomarker descriptions as in Figure S3.


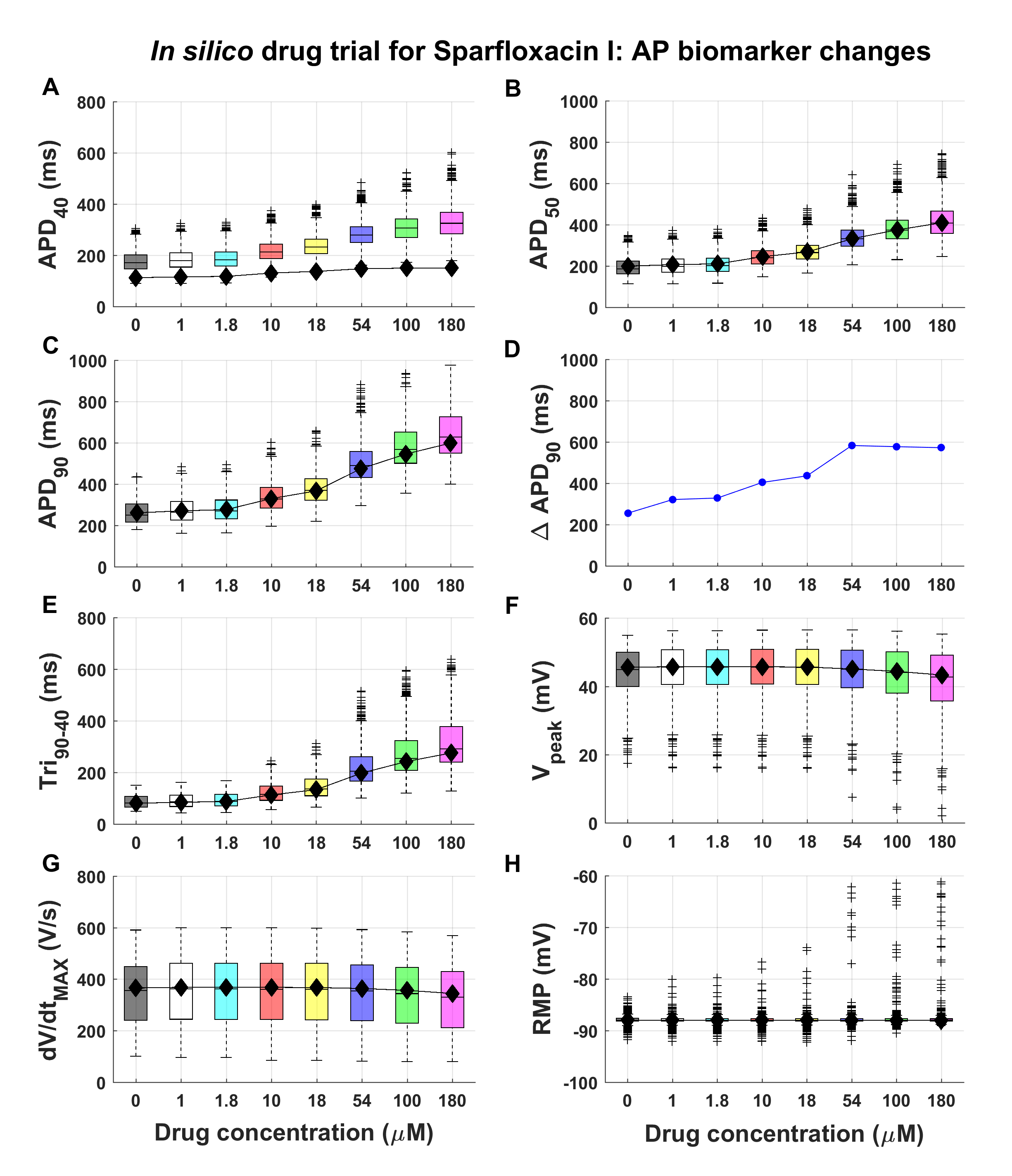


**Figure S27**. Sparfloxacin I effect on 8 AP biomarkers. Results are presented as boxplots showing the AP biomarker distributions in the population of human ventricular models, while the results for the baseline ORd model are shown as filled black diamonds. Boxplot and AP biomarker descriptions as in Figure S3.


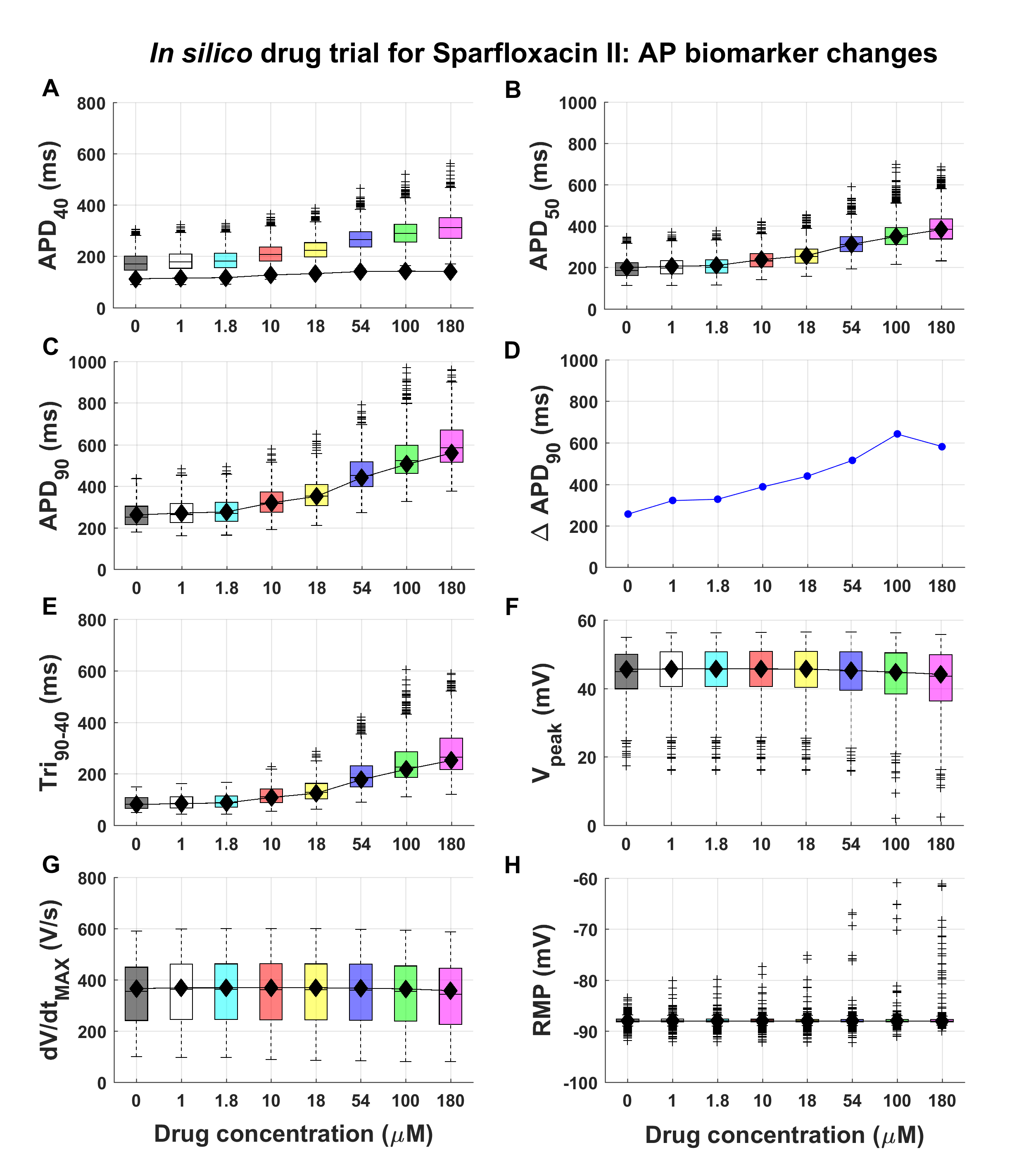


**Figure S28**. Sparfloxacin II effect on 8 AP biomarkers. Results are presented as boxplots showing the AP biomarker distributions in the population of human ventricular models, while the results for the baseline ORd model are shown as filled black diamonds. Boxplot and AP biomarker descriptions as in Figure S3.


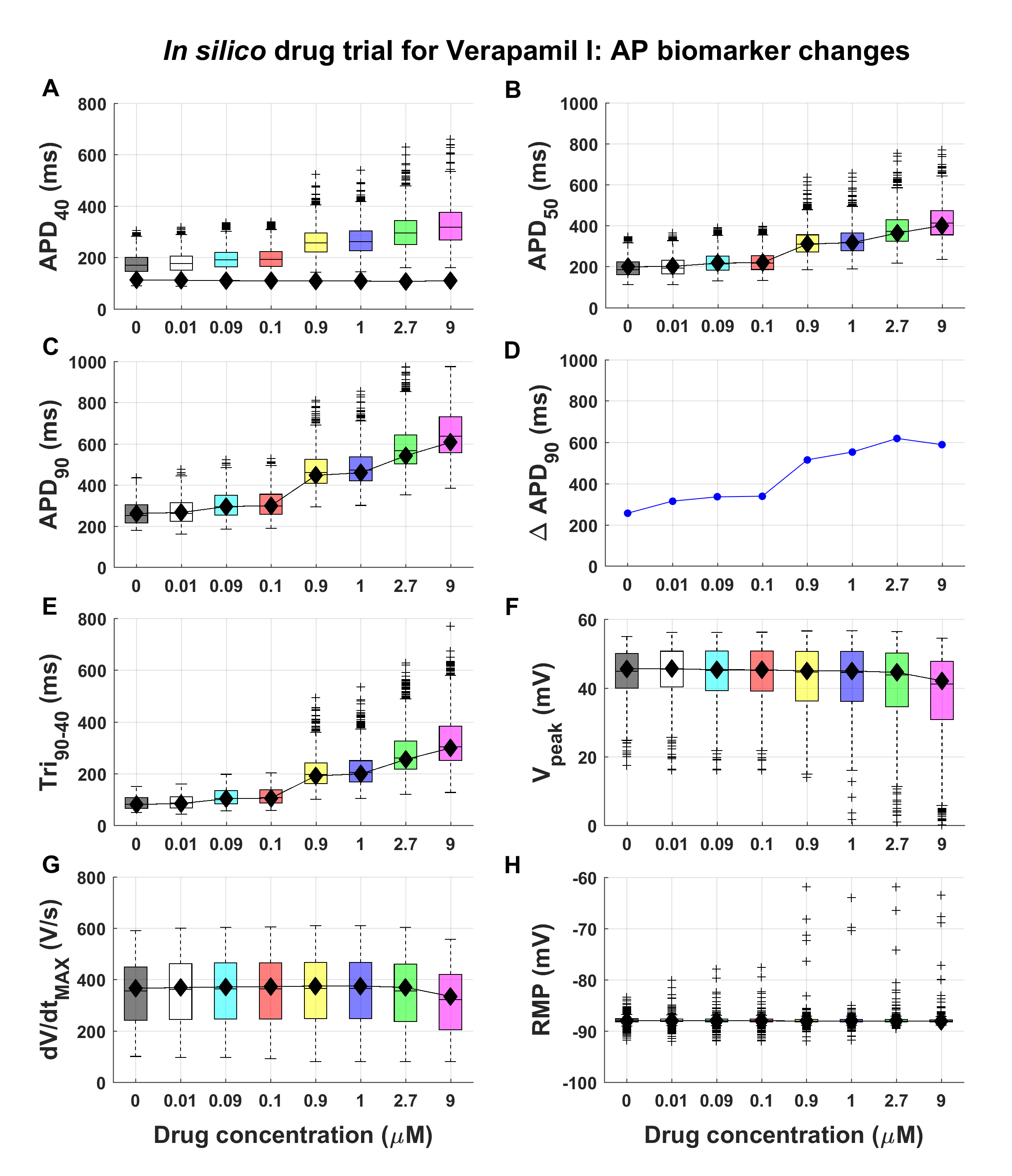


**Figure S29**. Verapamil I effect on 8 AP biomarkers. Results are presented as boxplots showing the AP biomarker distributions in the population of human ventricular models, while the results for the baseline ORd model are shown as filled black diamonds. Boxplot and AP biomarker descriptions as in Figure S3.


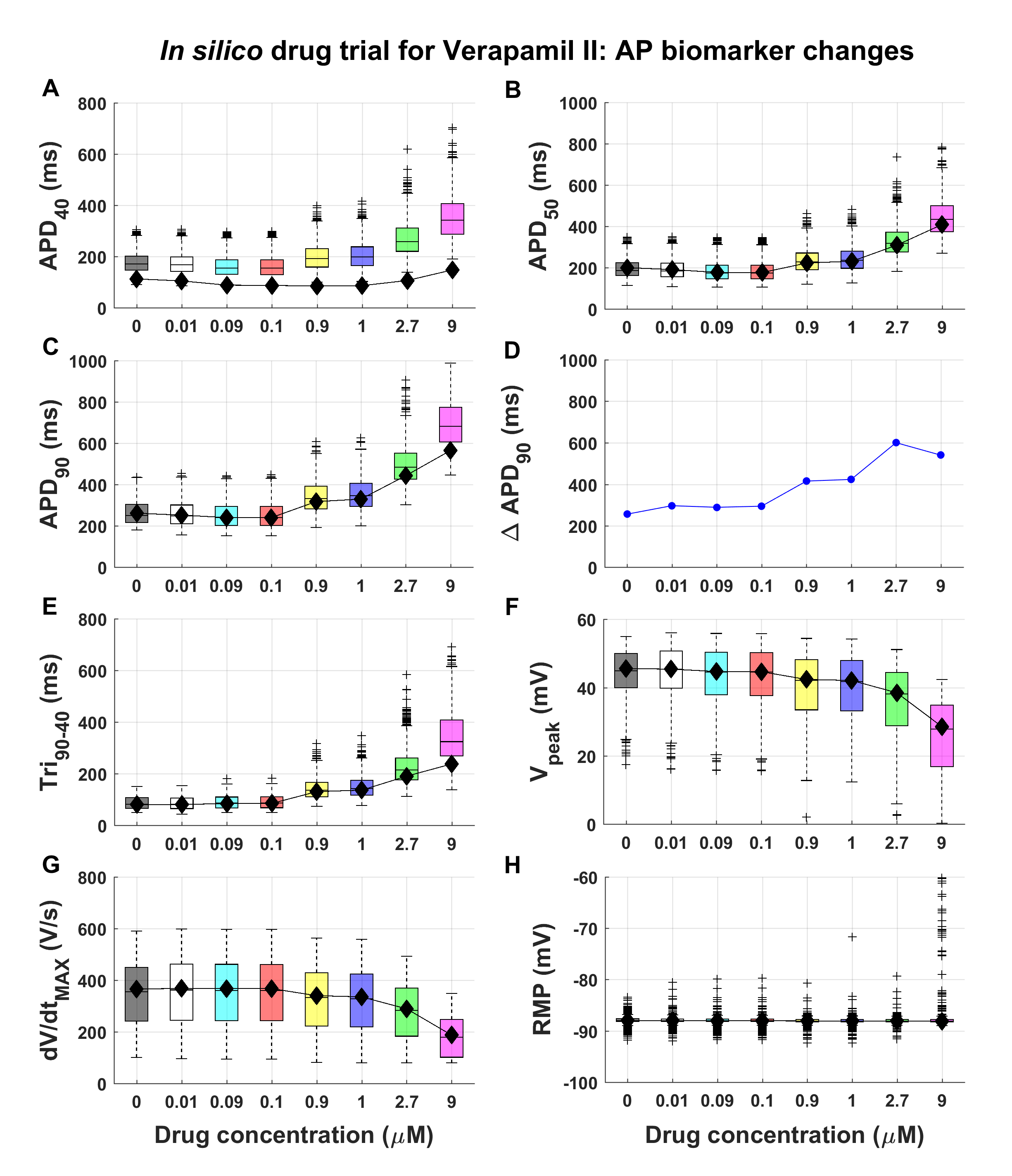


**Figure S30**. Verapamil II effect on 8 AP biomarkers. Results are presented as boxplots showing the AP biomarker distributions in the population of human ventricular models, while the results for the baseline ORd model are shown as filled black diamonds. Boxplot and AP biomarker descriptions as in Figure S3.


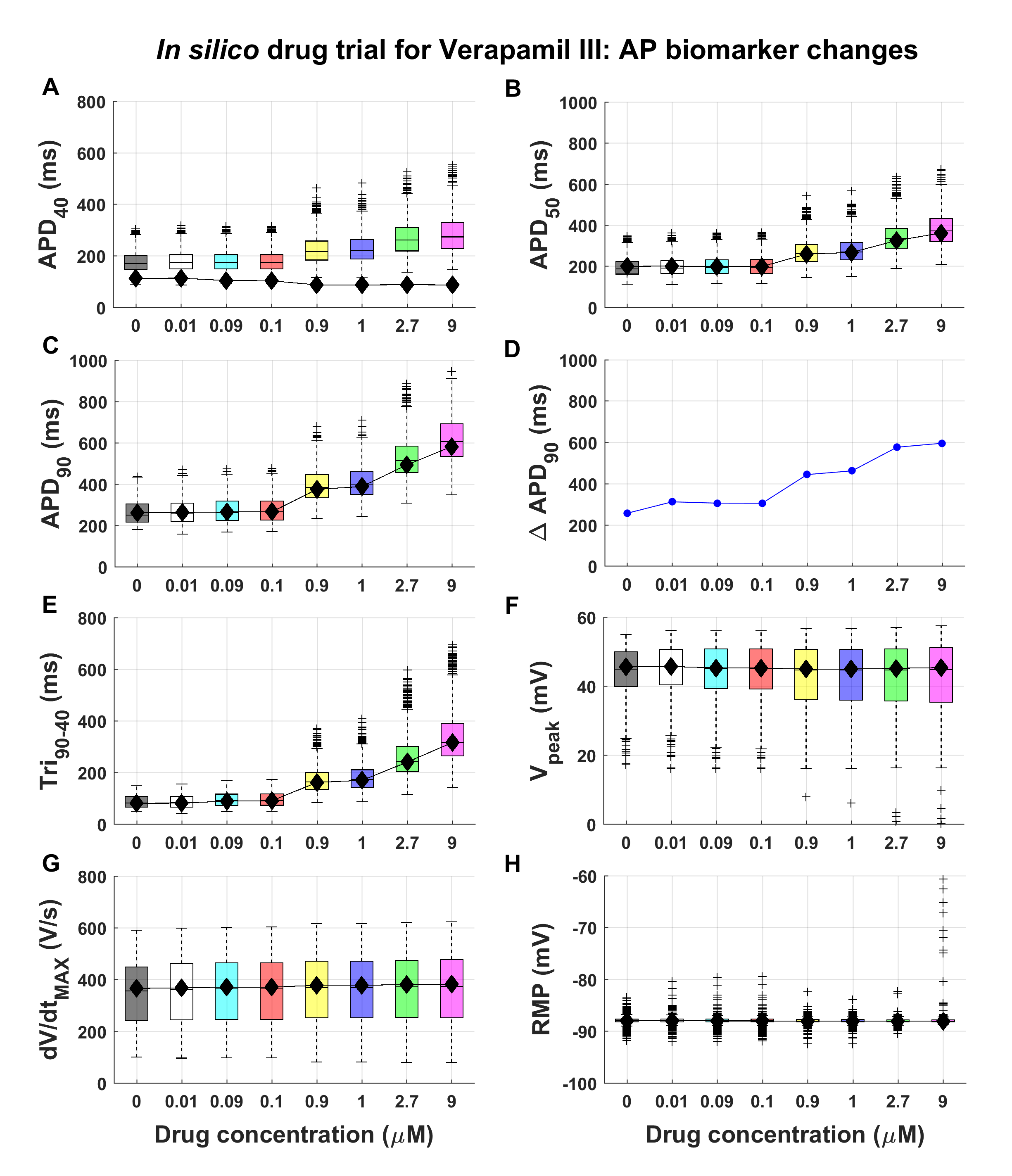


**Figure S31**. Verapamil III effect on 8 AP biomarkers. Results are presented as boxplots showing the AP biomarker distributions in the population of human ventricular models, while the results for the baseline ORd model are shown as filled black diamonds. Boxplot and AP biomarker descriptions as in Figure S3.

**
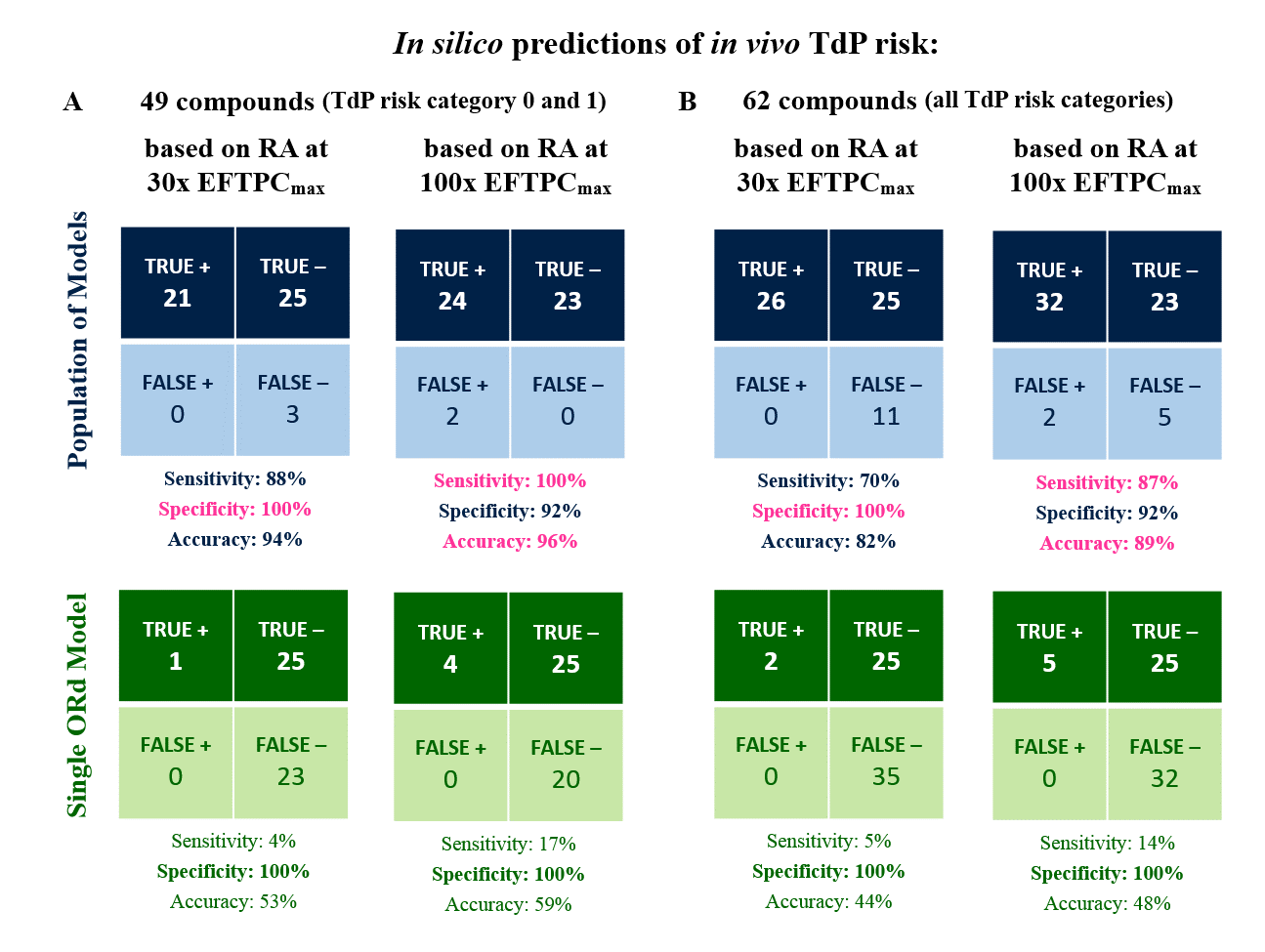
**

**Figure S32**. Comparison of the RA-based classification results obtained for concentration up to 30x and 100x EFTPC_max_, for the 49 compounds belonging to TdP risk category 0 and 1 **(A)** and for all the 62 tested compounds **(B)**. In each panel, predictions based on RA occurrence for concentrations up to 30x EFTPC_max_ (1^st^ column) are compared against the ones obtained for concentrations up to 100x EFTPC_max_ (2^nd^ column). Results obtained using the population of models (top half) are compared against the ones for the baseline ORd model (bottom half). High sensitivity/specificity/accuracy (>80%) are shown in bold, and the best results for each concentration are highlighted in pink. When the maximum tested concentration is higher, sensitivity increases, while specificity decreases. Overall, the accuracy is higher when considering 100x EFTPC_max_.

**
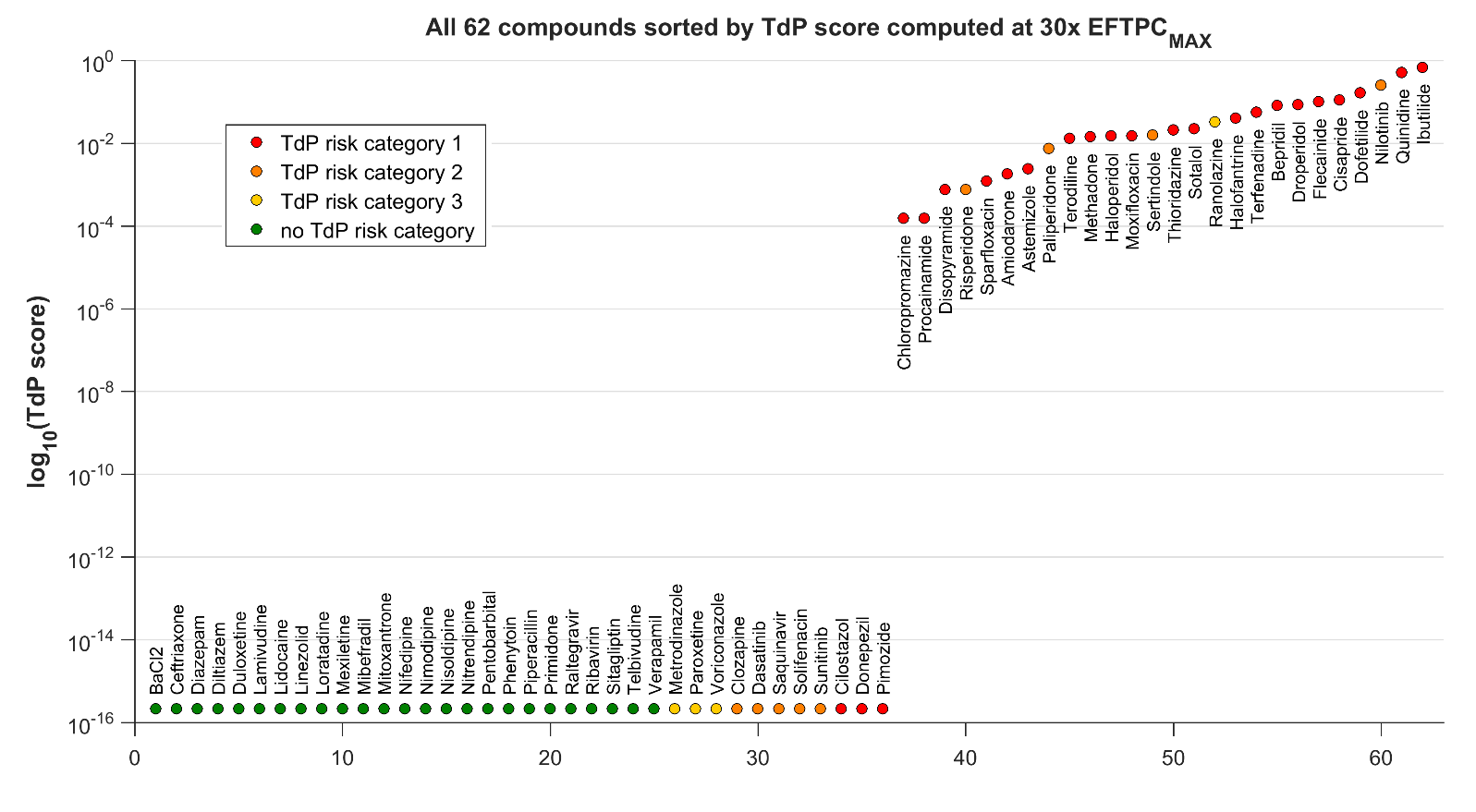
**


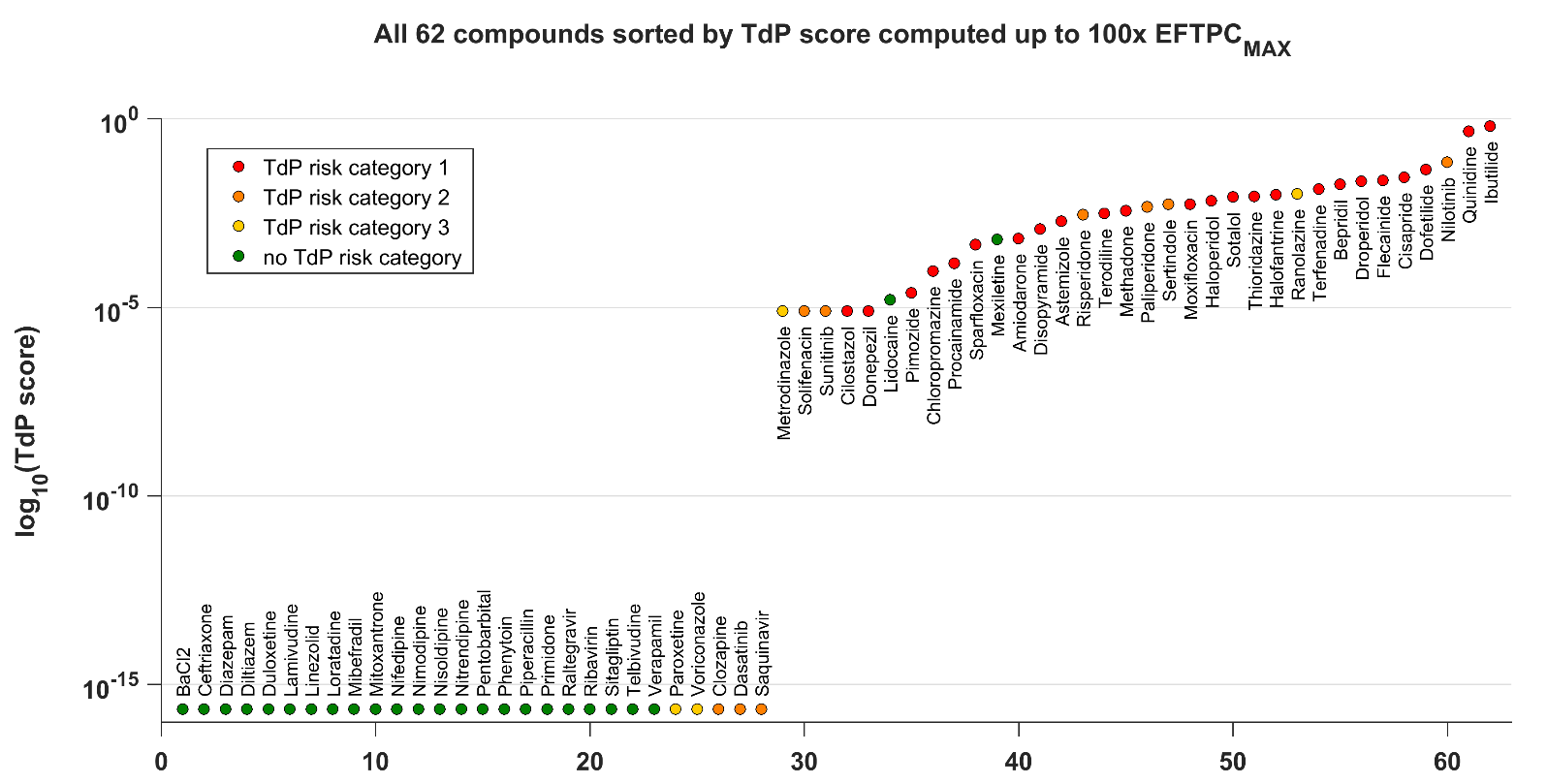


**Figure S33**. Comparison of the TdP score obtained for classification results obtained for concentration up to 30x (top panel) and 100x EFTPC_max_ (bottom panel). Concentration-dependent changes in the TdP score reflects the changes in sensitivity/specificity reported in Figure S32.

**Supplementary References**

Brennan, T., Fink, M., and Rodriguez, B. (2009). Multiscale modelling of drug-induced effects on cardiac electrophysiological activity. *Eur. J. Pharm. Sci.* 36, 62–77. doi:10.1016/j.ejps.2008.09.013.

Britton, O. J., Bueno-Orovio, A., Van Ammel, K., Lu, H. R., Towart, R., Gallacher, D. J., et al. (2013). Experimentally calibrated population of models predicts and explains intersubject variability in cardiac cellular electrophysiology. *Proc. Natl. Acad. Sci.* 110, E2098–E2105. doi:10.1073/pnas.1304382110.

Britton, O. J., Bueno-Orovio, A., Virág, L., Varró, A., and Rodriguez, B. (2017). The Electrogenic Na+/K+ pump is a Key Determinant of Repolarization Abnormality Susceptibility in Human Ventricular Cardiomyocytes: A Population-Based Simulation Study. *Front. Physiol.* 8, 278. doi:10.3389/FPHYS.2017.00278.

Clayton, R. H., Bernus, O., Cherry, E. M., Dierckx, H., Fenton, F. H., Mirabella, L., et al. (2011). Models of cardiac tissue electrophysiology: Progress, challenges and open questions. *Prog. Biophys. Mol. Biol.* 104, 22–48. doi:10.1016/j.pbiomolbio.2010.05.008.

Gemmell, P., Burrage, K., Rodriguez, B., and Quinn, T. A. (2014). Population of computational rabbit-specific ventricular action potential models for investigating sources of variability in cellular repolarisation. *PLoS One* 9, e90112. doi:10.1371/journal.pone.0090112.

Liberos, A., Bueno-Orovio, A., Rodrigo, M., Ravens, U., Hernandez-Romero, I., Fernandez-Aviles, F., et al. (2016). Balance between sodium and calcium currents underlying chronic atrial fibrillation termination: An in silico intersubject variability study. *Hear. Rhythm* 13, 2358–2365. doi:10.1016/j.hrthm.2016.08.028.

Muszkiewicz, A., Britton, O. J., Gemmell, P., Passini, E., Sánchez, C., Zhou, X., et al. (2016). Variability in cardiac electrophysiology: Using experimentally-calibrated populations of models to move beyond the single virtual physiological human paradigm. *Prog. Biophys. Mol. Biol.* 120, 115–127. doi:10.1016/j.pbiomolbio.2015.12.002.

O’Hara, T., Virág, L., Varró, A., and Rudy, Y. (2011). Simulation of the undiseased human cardiac ventricular action potential: Model formulation and experimental validation. *PLoS Comput. Biol.* 7, e1002061. doi:10.1371/journal.pcbi.1002061.

Passini, E., Mincholé, A., Coppini, R., Cerbai, E., Rodriguez, B., Severi, S., et al. (2016). Mechanisms of pro-arrhythmic abnormalities in ventricular repolarisation and anti-arrhythmic therapies in human hypertrophic cardiomyopathy. *J. Mol. Cell. Cardiol.* 96, 72–81. doi:10.1016/j.yjmcc.2015.09.003.

Reilly, S. N., Liu, X., Carnicer, R., Recalde, A., Muszkiewicz, A., Jayaram, R., et al. (2016). Up-regulation of miR-31 in human atrial fibrillation begets the arrhythmia by depleting dystrophin and neuronal nitric oxide synthase. *Sci. Transl. Med.* 8, 340ra74. doi:10.1126/scitranslmed.aac4296.

Sanchez, C., Bueno-Orovio, A., Wettwer, E., Loose, S., Simon, J., Ravens, U., et al. (2014). Inter-subject variability in human atrial action potential in sinus rhythm versus chronic atrial fibrillation. *PLoS One* 9, e105897. doi:10.1371/journal.pone.0105897.

Shampine, L. F., and Reichelt, M. W. (1997). The MATLAB ODE Suite. *SIAM J. Sci. Comput.* 18, 1–22. doi:10.1137/S1064827594276424.

Zhou, X., Bueno-Orovio, A., Orini, M., Hanson, B., Hayward, M., Taggart, P., et al. (2016). In Vivo and in Silico Investigation into Mechanisms of Frequency Dependence of Repolarization Alternans in Human Ventricular Cardiomyocytes. *Circ. Res.* 118, 266–278. doi:10.1161/CIRCRESAHA.115.307836.
